# Compliant rolling-contact architected materials for shape reconfigurability

**DOI:** 10.1038/s41467-018-07073-5

**Published:** 2018-11-02

**Authors:** Lucas A. Shaw, Samira Chizari, Matthew Dotson, Yuanping Song, Jonathan B. Hopkins

**Affiliations:** Mechanical and Aerospace Engineering, University of California, Los Angeles, Los Angeles, CA 90095 USA

## Abstract

Architected materials can achieve impressive shape-changing capabilities according to how their microarchitecture is engineered. Here we introduce an approach for dramatically advancing such capabilities by utilizing wrapped flexure straps to guide the rolling motions of tightly packed micro-cams that constitute the material’s microarchitecture. This approach enables high shape-morphing versatility and extreme ranges of deformation without accruing appreciable increases in strain energy or internal stress. Two-dimensional and three-dimensional macroscale prototypes are demonstrated, and the analytical theory necessary to design the proposed materials is provided and packaged as a software tool. An approach that combines two-photon stereolithography and scanning holographic optical tweezers is demonstrated to enable the fabrication of the proposed materials at their intended microscale.

## Abstract (web summary)

The authors introduce a compliant rolling-contact architected material as a class of shape reconfigurable mechanical metamaterial. They also devise an approach that combines two photon stereolithography and scanning holographic optical tweezers, which allows them to fabricate these architected materials on the microscale.

## Introduction

**Fig. 1:**
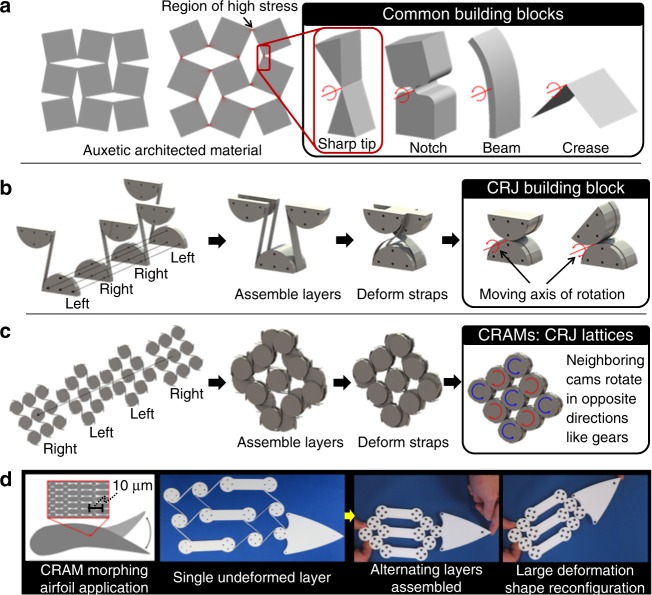
Introduction to compliant rolling-contact architected materials (CRAMS). **a** An auxetic architected material and other common building blocks used within existing shape-reconfigurable examples. **b** Compliant rolling-contact joint (CRJ) proposed as the building block for enabling the proposed CRAM lattices. **c** A square-tessellated CRAM example. **d** A shape-morphing airfoil consisting of differently shaped cams arranged in an aperiodic way

The underlying reason why architected materials (a.k.a. mechanical metamaterials) can achieve larger changes in shape than typical homogeneous materials is that their microarchitecture violates the affine assumption by producing nonuniformly distributed regions of deformation^1^. The auxetic architected material^2,3^ shown in Fig. 1a, for example, achieves its extreme expansion because its deformations are localized at sharp-tip hinges. Inspired by rigid-link mechanisms^1,4^, origami^5–7^, kirigami^8,9^, and multi-stable snapping structures^10–13^, engineers have utilized additional building blocks (e.g., notch flexures, buckled beams, and creases shown in Fig. 1a) to create metamaterials that achieve even more advanced changes in shape. Unfortunately, however, the building blocks used to achieve these capabilities increase in stress and strain energy as they are deformed thus limiting the material’s elastic range of motion and producing unwanted resistance in the direction of the desired deformations. Moreover, the small geometric features that enable the performance of these building blocks lower the stiffness of the bulk material in directions that are not desired and render the material susceptible to failure due to fatigue or yielding at the localized regions of high stress.

These shortcomings can be avoided by implementing compliant rolling-contact joints (CRJs)^14,15^ as an alternative building block within advanced shape-reconfigurable lattices called compliant rolling-contact architected materials (CRAMs). Traditional CRJs consist of four identical layers of half-cylinder cams joined together by initially straight flexure straps (Fig. 1b). The joint is created when the layers are assembled in the alternating sequence shown (i.e., left, right, right, left) and the flexure straps are deformed until both cams are aligned and joined together. Compared to other compliant-joint building blocks (e.g., Fig. 1a), the ratios of the resulting joint’s stiffness along its constrained directions to the angular stiffness about its moving rotational axis are extreme. CRJs can be designed to maintain constant levels of strain energy as they rotate and can thus theoretically achieve zero angular stiffness if their straps (i) are the exact length required to prevent them from becoming loose or being stretched when the joint is assembled, (ii) are fabricated to be perfectly straight with no variation in their geometry or constituent properties, (iii) are deformed over perfectly circular cylinders, and (iv) successfully guide the cylinders with perfect rolling-contact motion without stretching or slipping. These conditions also ensure that internal stress does not increase as the joint is rotated over its full range of almost 360^o^ (both statements are proven in Supplementary Note 1). In practice, however, such conditions are impossible to perfectly satisfy but even poorly made CRJs can approach these ideal behaviors more closely than alternative building block options (e.g., Fig. 1a).

The first known instantiation of rigid bodies constrained by crisscrossing straps similar to those of CRJs is the Jacob’s ladder toy of ancient origin^16^. The first published engineering mechanism, however, that used straps wrapped around cylindrical cams to guide rolling motions is the Rolamite mechanism^17,18^. This mechanism quickly evolved into the first rolling-contact joint that used crisscrossing straps to guide rotational motions between differently shaped cams^19,20^. The CRJ of Fig. 1b was then created^14,15^ with the realization that the straps and rigid cams could be made as monolithic layers, which could be deformed and assembled together. Although strap-based rolling-contact joints have been used to enable robotic hands^21^, prosthetic knees^22^, laparoscopic graspers^23^, gravity balancers^24^, origami-inspired deployable joints^25^, and self-deployable locking hinges^26^, this paper is the first to propose using this type of joint within lattices for creating architected materials (i.e., CRAMs).

Similar to CRJs, the proposed CRAMs consist of identical but differently oriented layers of cams joined together by initially straight flexure straps in a lattice (e.g., Fig. 1c). Once deformed and assembled, the resulting architectures share similar kinematics to closed-chains of gears that roll along each other’s contours to achieve large system-level shape changes while exhibiting the benefits of CRJs. Although others have proposed gear-based metamaterials for different purposes^27^, CRAMs do not require their cams to be mounted on underlying links and their pre-stretched straps prevent backlash, which occurs between the teeth of imperfectly aligned gears.

The unique properties of CRAMs enable advanced applications. Suppose, for example, stiff but lightweight aircraft wings were desired that could be actuated with minimal energy to alter their shape over a prescribed deformation for improving flight maneuverability and fuel efficiency (Fig. 1d). If aperiodic arrangements of differently shaped micro-cams could be packed within a single degree-of-freedom CRAM, various cross-sections could be combined to create such wings. These wings would require no outer covering since their individual cams would be small enough to approximate a smooth surface. A crude macroscale prototype is provided to demonstrate this concept using only a few aperiodic cams (Fig. 1d). Additionally, since CRAMs consist of tightly packed micro-cams that exhibit highly nonlinear stiffness properties along their constrained directions due to their circular shape, they could enable advanced micro-granular crystals^28^ that utilize shape reconfigurability to control the propagation of stress waves within their lattice. Such reconfigurable crystals could facilitate tunable acoustic lenses, sound scramblers, and photonic crystals. Finally, since CRAMs typically generate more friction than other metamaterials that use traditional compliant joints due to the stretching of their straps, CRAMs could be used to dissipate energy for mitigating impacts, particularly if their cams were shaped like polygons, which would produce extra strap stretching and multi-stability^29,30^. This concept would also enable CRAMs to passively maintain their deformed shapes over many states of stability.

In this paper, we classify CRAMs according to the number of degrees of freedom (DOFs) achieved collectively by the CRJs that constitute their architecture. Two-dimensional (2D) and three-dimensional (3D) macroscale CRAM prototypes are fabricated and tested to demonstrate their shape reconfigurability. Two different ways straps can be wrapped within CRAMs are characterized in the context of practical design guidelines. The mathematical theory is introduced for modeling the full behavior of CRJs as a function of their geometric parameters. This theory is integrated into a software tool that predicts how CRAMs consisting of arbitrary tessellations of CRJs deform over large ranges to achieve bulk changes in shape. The theory is used to compare the lattice properties of square-tessellated CRAMs (Fig. 1c) with the properties of other common materials. Finally, an approach that combines two-photon stereolithography and scanning holographic optical tweezers is demonstrated for fabricating CRAMs at the microscale.

## Results

### CRAM tessellations and examples

**Fig. 2:**
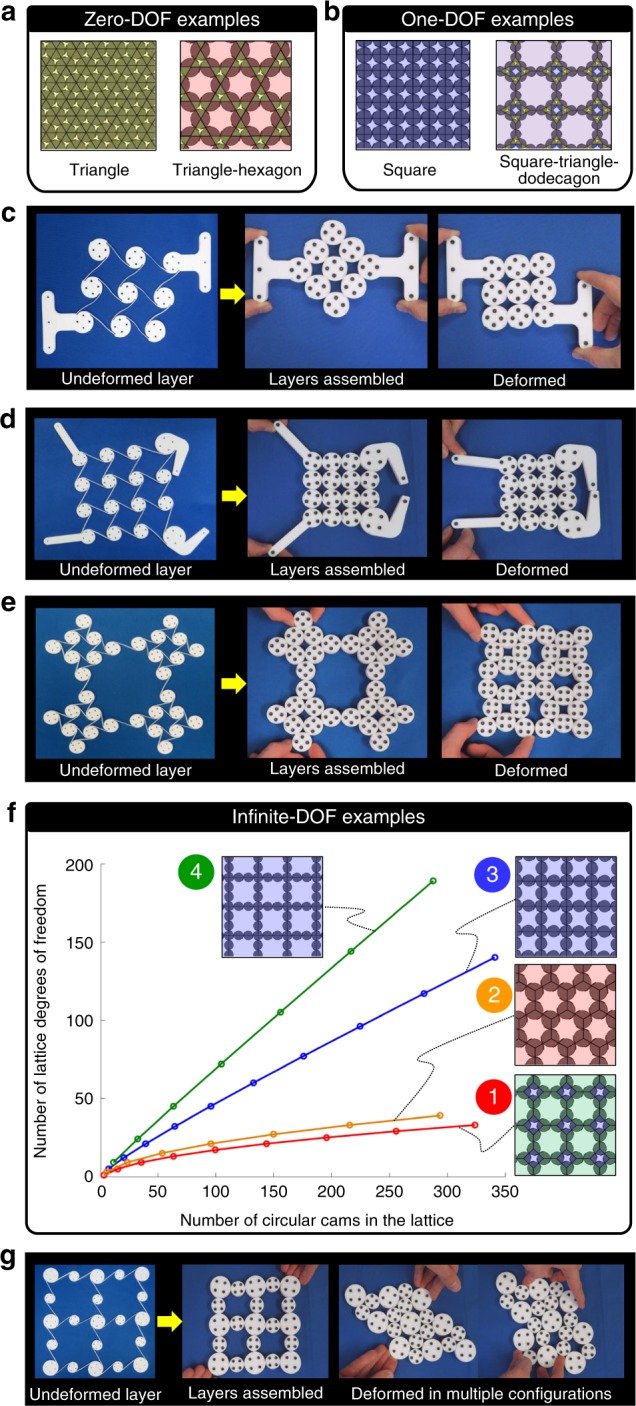
Circular cam tessellations and their degrees of freedom (DOFs). Example tessellations that achieve **a** zero DOFs and **b** one DOF. **c** One-DOF square-tessellated lattices can guide large motions and **d** can achieve large mechanical advantages. **e** One-DOF square–triangle–dodecagon tessellated lattices achieve auxetic behavior. **f** Some lattices achieve an increasing number of DOFs as their size grows. **g** Such infinite-DOF lattices can take on many shapes

The DOFs achieved by CRAMs are determined by how their cams are tessellated within their lattice and how their boundary is configured along their lattice’s edge. The theory necessary to calculate the number of DOFs achieved by general CRAMs of any tessellation and with any boundary configuration is provided in Methods. This theory was used to categorize three different kinds of CRAM tessellations according to their kinematic capabilities (e.g., zero-DOF, one-DOF, and infinite-DOF tessellations). Some tessellations, called zero-DOF tessellations, were observed to achieve zero DOFs regardless of their lattice size (e.g., triangle or triangle-hexagon tessellations of Fig. 2a). Other tessellations, called one-DOF tessellations, were observed to achieve one DOF regardless of their lattice size (e.g., square and square–triangle–dodecagon tessellations of Fig. 2b). Square tessellations from this category (e.g., Fig. 1c) could be used to maintain the parallel orientation between two bodies over a large range of motion (Fig. 2c) or they could be used to amplify or attenuate forces or displacements for other applications (e.g., the gripper in Fig. 2d). Square–triangle–dodecagon tessellations, also from the one-DOF category, could be used as large-range auxetic materials (Fig. 2e). Other tessellations, called infinite-DOF tessellations, approach an infinite number of DOFs as their lattice size grows indefinitely large. The plot of Fig. 2f demonstrates this observation for four infinite-DOF tessellation examples with different rates of DOF growth. Details about how their boundaries were configured as their lattices increased in size are provided in Methods. Note that the number of DOFs grows fastest for infinite-DOF lattices with cams that lie along the edges of the polygons that constitute their tessellation (e.g., those labeled 3 and 4) instead of just at their vertices as is the case for the slow growing square–octagon and hexagon tessellations labeled 1 and 2, respectively. Leveraging this observation, designers can generate infinite-DOF tessellations that achieve larger numbers of DOFs per lattice size so that the resulting CRAMs can achieve greater shape-morphing versatility. Note that the more DOFs a CRAM possesses, the more shapes it can assume. Thus the prototype of the infinite-DOF design labeled 3 in Fig. 2f can morph from its original shape into a variety of different shapes as shown in Fig. 2g.

**Fig. 3:**
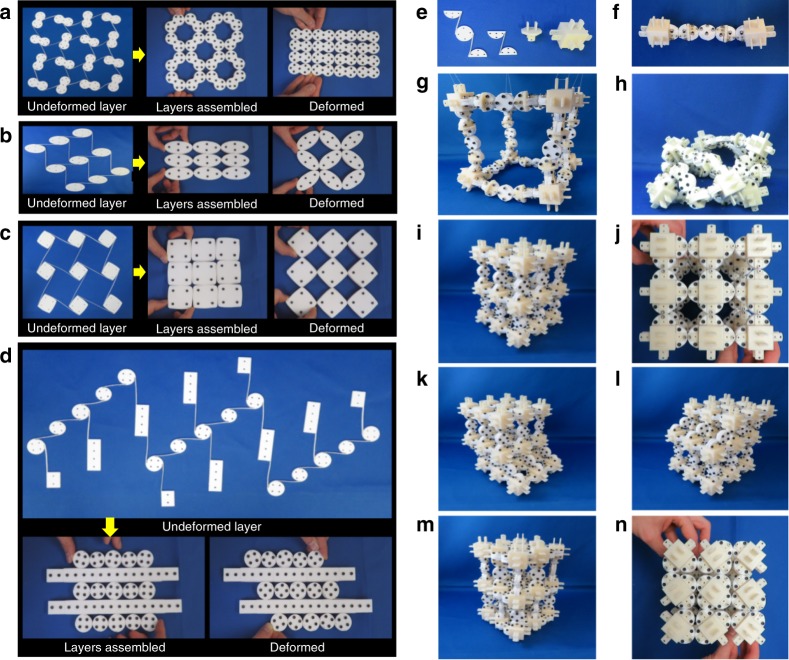
Other varieties of compliant rolling-contact architected materials (CRAMs). Shape morphing can be achieved using **a** peanut-shaped cams, **b** elliptical cams, and **c** square-shaped cams. **d** Shearing motions can be achieved by combining rectangular and circular cams. **e** Example parts can be fabricated to assemble **f** strings of CRJs that can be used to enable three-dimensional (3D) CRAMs. **g** A cube-shaped polyhedron of these strings could **h** tangle into many shapes. **i**, **j** Another 3D design that can **k**, **l** shear in tangential directions and exhibit **m**, **n** an auxetic behavior that stiffens those shearing directions

The cams that constitute CRAMs do not need to be circular. The periodic one-DOF lattices of Fig. 3a, b achieve positive Poisson’s ratios via peanut- and ellipse-shaped^31^ cams. The periodic one-DOF lattice of square-shaped cams in Fig. 3c achieve a negative Poisson’s ratio similar to the design of Fig. 1a but with almost twice the range of motion. Differently shaped cams can also be repeated throughout a lattice. The CRAM shown in Fig. 3d combines rectangular and circular cams to achieve a rack-and-pinion-inspired lattice that achieves pure shearing motion and possesses as many DOFs as there are rows of circular cams. Aperiodic CRAMs of differently shaped cams that do not repeat throughout the lattice’s geometry (e.g., Fig. 1d) show the most promise for achieving any prescribed change in shape.

CRAMs are also not restricted to 2D designs only. Curved sheets of the previously proposed 2D designs can be assembled if successive layers of cams are made slightly larger than prior layers. Additionally, cams from within the layers of different 2D designs can also be joined together using spacers to create quasi-3D materials that achieve desired cross-sectional shape changes. True-3D concepts can also be assembled using the example pieces shown in Fig. 3e. If the series of joints shown in Fig. 3f were assembled to form the edges of space-filling polyhedrons (e.g., the cube in Fig. 3g) within a lattice, string-like 3D CRAMs could be generated, which could be tangled to form many shapes (Fig. 3h). Other 3D configurations could be assembled to create lattices that achieve more orderly shape changes. Consider the 3D lattice shown from two perspectives in Fig. 3i, j. In the configuration shown, the lattice could shear in two orthogonal directions with near-zero stiffness (Fig. 3k, l). If, however, the lattice was actuated with its auxetic DOF, shown from two different perspectives in Fig. 3m, n, the lattice’s shear DOFs would substantially stiffen proportionately. Details pertaining to the fabrication of the macroscale prototypes shown in Figs. 1–3 are provided in Methods, and videos of the prototypes changing shape are provided in Supplementary Movie 1.

### Strap wrapping configurations

**Fig. 4:**
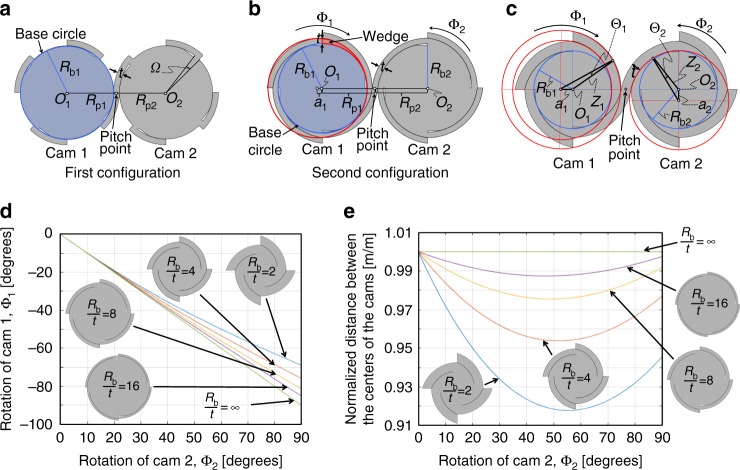
Two strap configurations. **a** First and **b** second way straps can be wrapped around neighboring cams. **c** Parameters used to model the kinematics of cams wrapped using the second configuration. **d** The rate that each cam rotates as well as **e** the distance between the cams’ centers will fluctuate for cams wrapped using the second configuration even for cam pairs that possess the same base-circle radius

Here we present two ways that straps can be wrapped around the cams that constitute general CRAMs for enabling their shape-reconfigurable properties. The first configuration (Fig. 4a) uses straps that are directly attached over an angle *Ω* to the perimeter of a circular cam’s base circle. The second configuration (Fig. 4b, c) uses wedge extensions that rise from each cam’s base-circle perimeter to the top surface of the straps to which the wedges connect. Note that the red circle that defines the outer perimeter of the example wedge labeled possesses a center that is offset a distance, *a*_1_, from the center, *O*_1_, of Cam 1’s blue base circle with radius, *R*_b1_. Note also that both circles are tangent to one another where the wedge rises from the base circle’s perimeter.

CRAMs wrapped using the first configuration (Fig. 4a) can exhibit the lowest achievable stiffness along their desired motion paths while also generating the least amount of friction because such CRAMs are wrapped the same way as traditional CRJs (e.g., Supplementary Fig. 1), and thus the conclusions derived in Supplementary Note 1 apply to these CRAMs. Note also that neighboring circular cams within CRAMs wrapped using the first configuration will rotate over angles that are linearly proportional to one another (i.e., Cam 1 will rotate *R*_p2_/*R*_p1_ times more than Cam 2 in the opposite direction, and *R*_p2_/*R*_p1_ remains constant regardless of either cam’s angular position). Moreover, the distance between the centers of their base circles (*O*_1_ and *O*_2_) will remain constant (i.e., *R*_b1_+*R*_b3_+*t*) due to geometric compatibility.

CRAMs wrapped using the second configuration of Fig. 4b, c will, however, not exhibit such simple kinematics. These CRAMs will, however, achieve more than twice as much deformation range compared to CRAMs wrapped using the first configuration. The reason is that straps wrapped using the second configuration can be much longer than straps wrapped using the first configuration because the straps are allowed to overlap as the cams roll. The downside to the second configuration is that even if both cams possess the same base-circle radius (i.e., *R*_b1_ = *R*_b2_), when one cam rotates at a constant rate, the neighboring cam will speed up or slow down due to the wedge on which the straps ride (Fig. 4d). Furthermore, the distance between the centers of the cams (i.e., *O*_1_ and *O*_2_) will also fluctuate as the cams rotate (Fig. 4e). Thus, if different layers of cams wrapped using the second configuration are oriented with the alternating sequence of Fig. 1c and their corresponding cams are joined together so that they are constrained to rotate the same amount and remain the same distance apart, the straps within the resulting CRAMs will be forced to stretch and slide as the cams rotate to accommodate these kinematic incompatibilities. Thus, as the cams rotate, their lattices will accrue strain energy, which will manifest as increasing actuation stiffness, and will dissipate heat, which will manifest as hysteresis. These unwanted effects can be mitigated if the strap thickness, *t*, is small with respect to each cam’s base-circle radius, *R*_b1_ and *R*_b2_. Note from the plots of Fig. 4d, e that, as the base-circle-radius-to-strap-thickness ratios increase to infinity, CRAMs that utilize the second configuration (Fig. 4b, c) approach the favorable behavior of CRAMs that utilize the first configuration (Fig. 4a) but still achieve more than twice as much rotational range. These plots can help designers know if their CRAM’s base-circle-to-strap-thickness ratio is high enough to permit the large-range benefits of the second wrapping configuration while sufficiently achieving the low stiffness and low friction characteristics of the first configuration. The theory used to generate the plots of Fig. 4d, e are provided in Methods.

### Design tool and theory validation

**Fig. 5:**
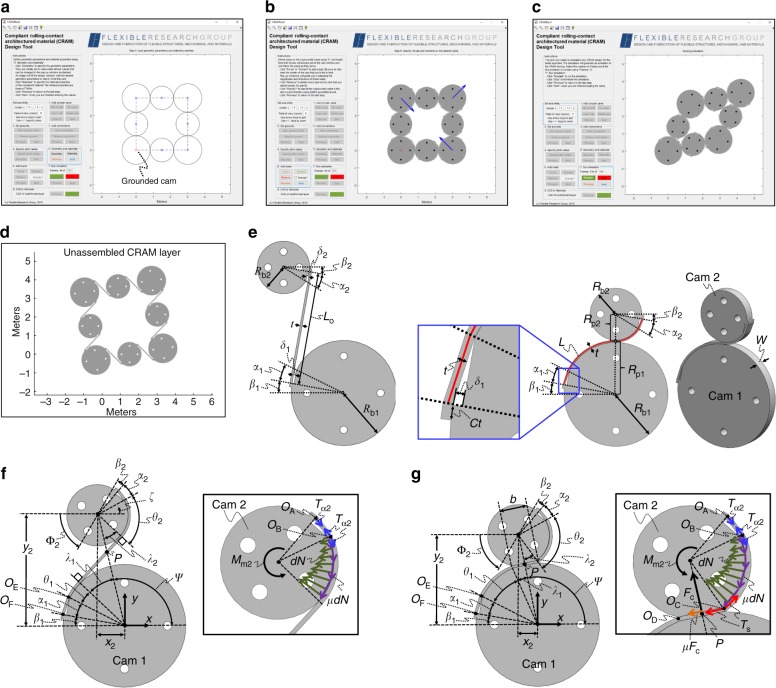
Software design tool enabled by the compliant rolling-contact joint (CRJ) modeling theory. **a** Cam locations, connections, and radii are specified by the user in the tool’s design window. **b** Loads are defined. **c** The design is simulated. **d** Its unassembled layer geometry is given. **e** Geometric parameters that define a single CRJ layer. Parameters that define the strap loads imparted on CRJ cams for general **f** tension and **g** compression scenarios

Although selecting the appropriate wrapping configuration is an important consideration during the design of CRAMs, the most difficult design challenges include (i) visualizing how the bulk material will deform for a given cam tessellation, (ii) sizing the straps within the chosen tessellation such that they are geometrically compatible while enabling the largest deformation range, and (iii) reverse engineering how each layer should be fabricated with straight straps once the final CRAM design is determined. No software currently exists for solving these issues, and current finite element packages are insufficient for simulating the friction-sensitive nonlinear behavior of these multi-layered pre-deformed lattices consisting of numerous cams. Additionally, fabricating prototypes to observe their behavior is expensive and time consuming. Thus we created an open-source MATLAB tool (see Supplementary Software 1) to facilitate the design of CRAMs. The tool begins by prompting the user to upload, enter, or click on locations within a scalable design window where cams are desired (Fig. 5a). It then prompts the user to define grounded cams from among those entered that will be held fixed. The user then chooses which cams she/he would like to join together using CRJs. The code then calculates the resulting radii of all the cams after the user enters the radius of one of them. The required parameters and constituent properties of the CRAM are then entered. The code then calculates the straps’ optimal lengths, displays the resulting design (Fig. 5b), calculates how many DOFs the design will achieve, and informs the user if the resulting design will yield when assembled. The user can then apply gravity or other desired forces and moments on desired cams, and the tool will generate an animated GIF of the resulting simulation (Fig. 5c). Finally, the user is provided with an image of how each layer of the final design should be fabricated with straight straps (Fig. 5d). A demo video of this tool is provided in Supplementary Movie 2.

The complete analytical theory required to enable this tool is detailed in Supplementary Notes 2 and 3. Although others have generated equations for approximating how CRJs respond to limited loading scenarios^32^, the equations in Supplementary Note 2 model the full nonlinear behavior of general CRJs for any loading scenario. The parameters used to define the geometry of each layer within a general CRJ are given in Fig. 5e. The parameters used to calculate the strap loads imposed on the cams within each CRJ layer for any tensile (Fig. 5f) or compressive (Fig. 5g) loading scenario are also given. Free body diagrams that show the loads within various portions of CRJ straps are provided in Supplementary Fig. 2. The tensile loading scenario (Fig. 5f) occurs when the straps that join the cams of a CRJ are stretched such that the cams are separated from each other. In this scenario, each strap within the CRJ imparts tensile, compressive, and frictional forces as well as a moment (Fig. 5f) on the cams that it joins to resist the external load that caused the cams to separate. The compressive loading scenario (Fig. 5g) occurs when the CRJ’s cams are pushed together and sandwich a flattened portion of their connecting straps. Although cams within this scenario can experience similar loads to those in the tensile loading scenario if their straps were sufficiently stretched when the CRJ was initially assembled, the compressive loading scenario is dominated by additional compressive forces caused primarily by Hertzian contact between the cams as well as other frictional forces (Fig. 5g). The theory required to apply the CRJ-modeling mathematics of Supplementary Note 2 to predict the behavior of general CRAMs consisting of CRJs tessellated together within arbitrary lattices is provided in Supplementary Note 3 along with the theory required to perform all other design capabilities achieved by Supplementary Software 1.

**Fig. 6:**
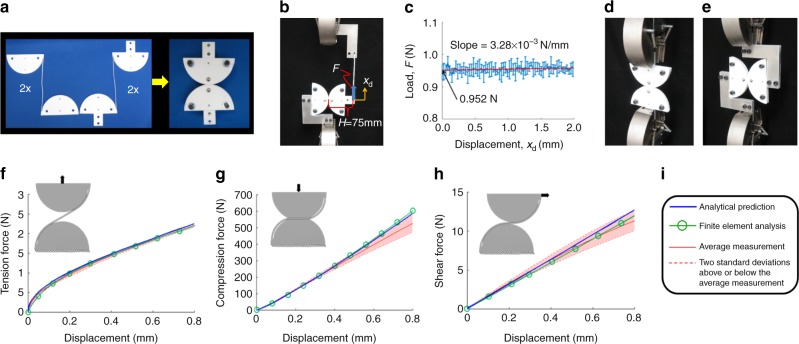
Experimental validation. **a** Fabricated four-layer compliant rolling-contact joint (CRJ). **b** Test set-up for measuring the angular stiffness of the CRJ and **c** results collected from five sets of data. Test setup for **d** tension, compression, and **e** shear. Finite element analysis (FEA) verification and experimental validation of the four-layer CRJ in **f** tension, **g** compression, and **h** shear. **i** Plot key

To validate the theory underlying the software tool, a four-layer CRJ made of Teflon was fabricated and assembled (Fig. 6a) using the material properties and geometric parameters described in Methods. Waterjet-cut aluminum fixtures were used in conjunction with a string to measure the CRJ’s angular stiffness as shown in the test set-up of Fig. 6b. The resulting Instron-generated data are provided in the plot of Fig. 6c. The angular stiffness of the CRJ was calculated by identifying the slope of the data’s line of best fit and multiplying this slope by *H*^2^, labeled in Fig. 6b. The resulting angular stiffness was determined to be 0.018 Nm rad^−1^. Five sets of data were collected to calculate the error bars shown in the plot of Fig. 6c. Note from these error bars that the predicted near-zero angular stiffness is experimentally validated. The same CRJ was also loaded in tension (Fig. 6d), compression (Fig. 6d), and shear (Fig. 6e) to validate the nonlinear stiffness of the joint’s constrained directions. Plots showing the resulting data compared against finite element analysis (FEA) and the analytically predicted force–displacement response of the CRJ in tension, compression, and shear are provided in Fig. 6f–h respectively. The key for each plot is provided in Fig. 6i. Additional details pertaining to how the experimental tests and FEA of these plots were performed are provided in Methods.

### CRAM material properties

**Fig. 7:**
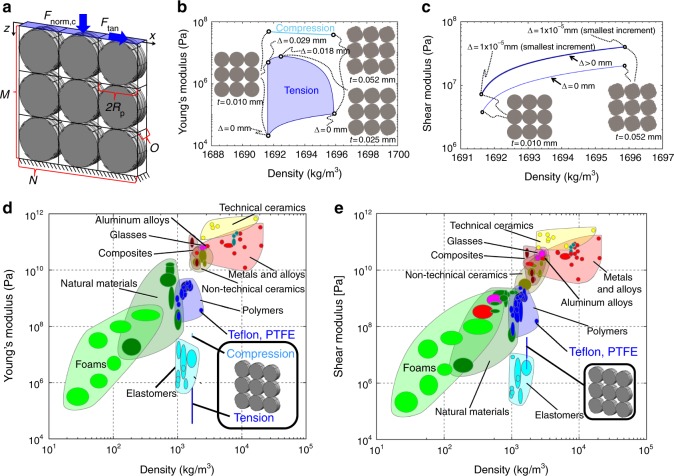
Properties of square-tessellated compliant rolling-contact architected materials (CRAMs) made of Teflon. **a** Parameters that define the geometry of square-tessellated CRAMs. **b** Performance regions for the compressive and tensile Young’s modulus versus density and **c** shear modulus versus density achieved by such CRAMs. Extreme CRAM examples are provided from along the boundaries of these regions. **d**, **e** The same performance regions plotted on Ashby plots to compare the properties of square-tessellated CRAMs made of Teflon with other common materials

The validated theory underlying the design tool can also be used to predict other properties achievable by general CRAMs beyond shape deformations. Here we provide an example to demonstrate how other system-level properties of square-tessellated CRAMs (e.g., Figs. 1c,  2b, c, and 7a) consisting of same-sized circular cams wrapped using the first configuration of Fig. 4a could be calculated. The bulk properties of interest here are the compressive, tensile, and shear moduli of such CRAM lattices as well as their density.

The compressive Young’s modulus, *E*_lattice,c_, of such CRAMs in the direction of the force labeled *F*_norm,c_ in Fig. 7a is defined as *E*_lattice,c_ = *σ*_lattice,c_/*ε*_lattice,c_ where the compressive stress imposed on the lattice is *σ*_lattice,c_ = *F*_norm,c_/(2*R*_p_*NWO*) and the resulting lattice strain is *ε*_lattice,c_ = *z*/(2*R*_p_*M*). Note that *F*_norm,c_ is a compressive force that is applied uniformly over the shaded surface shown in Fig. 7a, *z* is the resulting displacement of that surface, *R*_p_ is the common pitch-circle radius of the cams, *N* is the number of cam columns in the lattice, *M* is the number of cam rows, *O* is the number of layers that alternate their orientation according to the sequence shown in Fig. 1c, and *W* is the thickness of each layer. Thus another expression for the lattice’s compressive Young’s modulus is *E*_lattice,c_ = (*F*_norm,c_/*z*)(*M*/(*NWO*)). Note that since the lattice is a collection of CRJ layers arranged in various parallel and serial configurations and that each CRJ layer possesses a compressive stiffness, *k*_compression_, which can be derived using the theory of Supplementary Note 2 for modeling CRJs in any configuration, it can also be shown that (*F*_norm,c_/*z*) = (*k*_compression_*ON*)/*M*. Note that *k*_compression_ is the force-to-displacement ratio of a single CRJ layer in compression as shown in the image of Fig. 6g. Thus the most simplified expression of the lattice’s compressive Young’s modulus is *E*_lattice,c_ = *k*_compression_/*W*. A similar derivation can be used to show that the lattice’s tensile Young’s modulus is given by *E*_lattice,t_ = *k*_tension_/*W*, where *k*_tension_ is the tensile stiffness of a single CRJ layer in tension as shown in the image of Fig. 6f. If the force *F*_tan_, labeled in Fig. 7a, is used to replace *F*_norm,c_, and *x* is used to replace *z* in the previous derivation, the lattice’s shear modulus is proven to be *G*_lattice_ = *k*_shear_/*W*, where *k*_shear_ is the shear stiffness of a single CRJ layer as shown in the image of Fig. 6h. The stiffness values *k*_compression_, *k*_tension_, and *k*_shear_ can be derived using the theory of Supplementary Note 2. The density of square-tessellated CRAMs, *ρ*_lattice_, consisting of identical circular cams (i.e., *α*_*i*_ = *α*, *R*_b*i*_ = *R*_b_, and *R*_p*i*_ = *R*_p_) is given by $\rho _{{\mathrm{lattice}}} = \rho \left( {R_{\mathrm{b}}^2{\mathrm{\pi }} + t{\mathrm{\pi }}R_{\mathrm{p}} - 2R_{\mathrm{b}}^2\sin (\alpha )\left( {1 - \cos (\alpha )} \right)} \right)/\left( {2R_{\mathrm{b}} + t} \right)^2$ where *ρ* is the density of the constituent material, and all other geometric parameters specified are labeled in Fig. 5e and described in Supplementary Note 2. Note that none of the lattice properties, *E*_lattice,c_, *E*_lattice,t_, *G*_lattice_, or *ρ*_lattice_, are dependent on *N*, *M*, or *O*. Furthermore, none of these properties are appreciably affected by *W* or scale factor (i.e., if all the geometric parameters are multiplied by a common scale factor, the system-level properties remain largely unaffected). The only geometric parameters that appreciably affect the properties of square-tessellated CRAMs consisting of identical cams are (i) strap thickness, *t*, relative to base-circle radius and (ii) the amount that the strap is stretched when the CRJ is assembled, Δ. Thus the full performance regions of such CRAMs can be identified by calculating their lattice properties achieved from a complete sweep of these two parameters for a given base-circl radius of arbitrary size. Using the geometric parameters labeled in Fig. 5e, we arbitrarily set *R*_b_ = 0.5 mm and *W* = 0.25 mm and swept the parameters *t* and Δ. We set *δ*_1_ = *δ*_2_ = 0.01 mm because the smallest feature size that can be fabricated by our micro-fabrication approach is ~1% of 2*R*_b_. We set *β*_*i*_ = (π/4)+(*Ct/R*_b*i*_) because the lattices are square tessellations and *C*, labeled in Fig. 5e, is set to 1 so that the straps attach to their cams over a length of one strap thickness. The strap thickness, *t*, was swept using a resolution increment of 0.01 μm from the smallest feature size that can be fabricated (i.e., 0.01 mm) to the thickest amount that the straps can be without yielding when they are stretched and bent around their circular cams. The parameter Δ was swept using the same resolution increment of 0.01 μm from 0 mm to the largest bending thickness the fabricated straps could be without yielding when they are stretched and bent around their circular cams. Thus both parameters were constrained to satisfy $\sigma _{{\mathrm{y,t}}} \ge (E_{\mathrm{t}}\Delta /(L - \Delta )) + (E_{\mathrm{t}}t/(2R_{\mathrm{b}} + t))$, where *σ*_y,t_ is the tensile yield strength of the CRJ’s material, *E*_t_ is its tensile Young’s modulus, and the other geometric parameters are labeled in Fig. 5e and described in Supplementary Note 2.

Thus, by sweeping *t* and Δ in this way for the material properties of Teflon provided in Methods, the resulting performance regions of compressive, tensile, and shear moduli versus density were generated (Fig. 7b, c). Since *k*_compression_ and *k*_tension_ are theoretically zero before the lattice is strained a finite amount but increase rapidly for appreciable strains, the plotted values of *E*_lattice,c_ and *E*_lattice,t_ were calculated using a strain of 0.1% to provide a fair and practical comparison with the properties of other common materials. Since *k*_shear_, on the other hand, is already substantial before the lattice is strained, the plotted values of *G*_lattice_ were calculated using a zero strain. Note from Fig. 7b that the performance region of compressive Young’s modulus versus density is a simple curve because it is only dependent on *t*, whereas the performance region of tensile Young’s modulus versus density is an area because it is dependent on both *t* and Δ. Note also from Fig. 7c that the performance region of shear modulus versus density is both a curve when Δ = 0 and a thin area when Δ>0. Figure 7b, c also provide various versions of square-tessellated CRAMs from along the borders of the performance regions. The same regions are shown plotted again in Fig. 7d, e to compare them against the performance capabilities of common materials. The regions are shown compressed to vertical lines because the density axes of the plots use a log scale. Note that the theory introduced here for generating the boundaries of such performance regions can also be used to optimize the geometric parameters of CRAM designs such that they achieve desired material property combinations while also achieving desired shape deformations.

Although the plots of Fig. 7d, e reveal that the stiffness and density properties achieved by square-tessellated CRAMs made of Teflon are not as well suited for high-stiffness-to-low-density applications as most other common materials, other CRAM tessellations made of different constituent materials (e.g., metals) could be made to populate more practical regions of the plot. It is important to note that the purpose of providing the plots of Fig. 7d, e is not to compare the stiffness-to-weight ratios of CRAMs with common materials as much as it is to demonstrate the ability of this paper’s theory to generate performance regions of general CRAM tessellations made of any constituent material on a variety of useful Ashby plots. The ability to generate such regions for general CRAM scenarios is important if designers wish to compare other practical mechanical properties exhibited by the CRAMs they design for applications that require demanding shape reconfigurability.

The plots of Fig. 7d, e also demonstrate that the stiffness values of an optimized square-tessellated CRAM averaged along its principle directions will decrease by approximately one order of magnitude compared to its constituent material to enable the desired shape-morphing capabilities achieved by its architecture. For material applications that require extreme shape reconfigurability, such a loss of stiffness is an inevitable but likely an acceptable consequence when considering alternative options. The decrease in constituent material stiffness exhibited by other metamaterials with comparable ranges of shape reconfigurability such as origami^5–7^ and kirigami^8,9^ are typically significantly larger and thus less suited for practical applications.

### Fabrication of microscale CRAMs

**Fig. 8:**
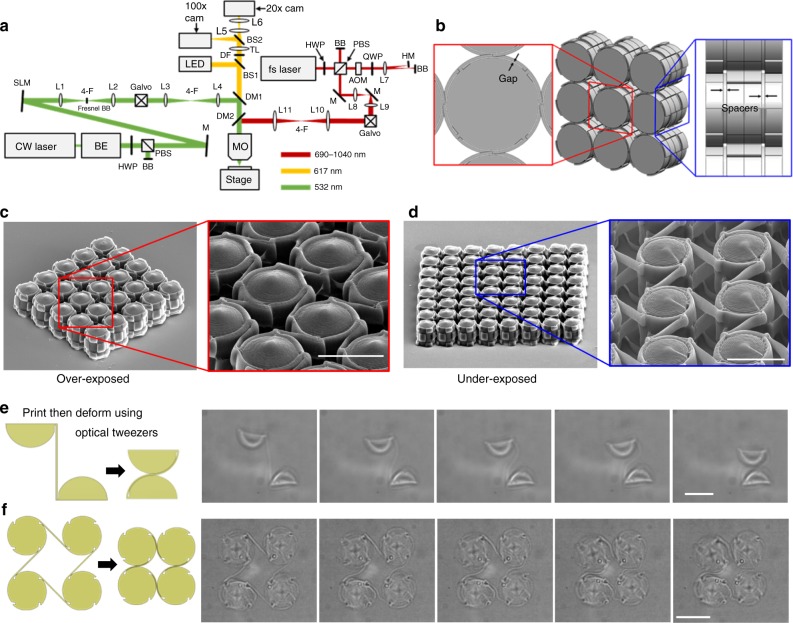
Approach that enables the microfabrication of compliant rolling-contact architected materials (CRAMs). **a** Our system can be used to directly print CRAM lattices with already curled straps as long as **b** gaps and spacers exist in the design. Such lattices fail to function, however, when they are slightly **c** over-exposed or **d** under-exposed. Our system can also use scanning holographic optical tweezers to deform the flexure straps after they have been printed straight to fabricate **e** compliant rolling-contact joints (CRJs) and **f** CRAMs with stored strain energy so they can achieve their intended properties (scale bar in **c** and **d** 10 μm, **e** 20 μm, **f** 30 μm)

An approach that combines the utility of two-photon stereolithography^33^ (2PS) with scanning holographic optical tweezers^34^ (SHOT) has been created and demonstrated for enabling the fabrication of CRAMs on the microscale. The system is shown in Fig. 8a but its subcomponents are detailed in Methods.

This system could fabricate CRAMs in two ways. The first way, which could be replicated using other existing 2PS systems, is to print CRAM lattices with straps that are already curled around their cams in an initial strain-free state using 2PS only. Such CRAMs would, however, need to be designed with specialized gaps between their straps and cams as well as spacers between their layers (Fig. 8b) to prevent the resulting lattices from binding due to straps that are fused to their cams. Even if such designs could be perfectly fabricated, however, these gaps would produce slop between the cams, which would drastically lower the CRAM’s compressive and tensile Young’s moduli. Moreover, an unwanted actuation stiffness would exist, which would grow as the cams rotate because their straps would be fabricated with an initially curved contour in a strain-free state. It is also difficult to fabricate such CRAMs as intended since they either end up with straps that fuse to their cams if they are slightly over-exposed to the laser (Fig. 8c) or the straps unwrap if they are under-exposed (Fig. 8d). Additional photos of CRAMs fabricated using the 2PS portion of our system are provided in Supplementary Fig. 3a-f.

The second way our system could fabricate CRAMs is currently the only existing way that such CRAMs could be fabricated with tightly wrapped straps that initially store and maintain strain energy over their full range. With this method, the 2PS portion of the system first uses a femtosecond laser to print successive layers of cams connected together with straight straps by curing desired regions within a polymer bath. The system then uses its integrated SHOT capabilities to generate multiple optical traps that simultaneously impart the necessary loads at different locations on each layer to strategically deform them as they freely float in the original polymer medium. This process is demonstrated for CRJ and square-tessellated CRAM layers in Fig. 8e, f, respectively. These layers were observed to successfully store strain energy because they would unwrap and return to their original shape when the optical traps holding them together were removed. Although others have used a single optical trap to deform grounded structures printed using an independent 2PS system^35^, our system is the first of its kind that can generate and independently coordinate multiple optical traps simultaneously over a large working area to impart forces and moments on free-floating structures printed using 2PS in the same machine for deforming and assembling the structures as desired. We have also demonstrated the ability to print, deform, and assemble two alternating layers of cams on top of each other that are half the size of those shown in Fig. 8f. To do this, the first deformed layer is pinned to the bottom of the substrate and a fence is printed around it so that the straps remain deformed without being held by optical traps. The second layer is then printed and deformed in place above it (see Supplementary Movie 3).

## Discussion

A class of architected materials (i.e., CRAMs) has been created that consists of micro-cams wrapped together by flexure straps, which guide internal rolling motions to achieve bulk lattice shape changes. CRAMs show promise for achieving extreme morphing capabilities without storing additional strain energy or increasing their internal stress as they are actuated. The theory necessary to model the elastomechanic behavior of general CRAMs and to rapidly analyze their kinematics over large deformations for desired actuation scenarios is provided. This theory has been integrated within an advanced software tool for facilitating the general design of CRAMs. Additionally, since CRAMs require that their layers be deformed and assembled after they are fabricated, a hybrid 2PS and SHOT approach was created to enable their fabrication on the microscale. These advances lay a strong foundation for the additional study and practical implementation of such materials.

## Methods

### Calculating the mobility of CRAM tessellations

The theory of interconnected-hybrid-flexure-system mobility analysis^36^ was adapted to enable designers to calculate the number of DOFs achieved by general CRAM tessellations of any boundary configuration. To demonstrate the approach, consider the simple CRAM example shown in Supplementary Fig. 4a. The first step of the approach is to draw a graph of the CRAM system using nodes that represent the lattice’s cams, labeled *ci* in the figure, and arrows that represent the rotational DOFs permitted by each joint at their pitch point, labeled **T**_*j*_. The directions of the graph’s arrows are arbitrary, but they establish an important convention that must be maintained for future steps. The second step is to create the graph’s incidence matrix, [Inc], according to previously published instructions^36^. For the example case study, the incidence matrix is

1 $$\left[ {\mathrm{Inc}} \right] = \left[ {\begin{array}{*{20}{c}} { - 1} & 1 & 0 & 0 \\ 0 & { - 1} & 1 & 0 \\ 0 & 0 & 1 & { - 1} \\ { - 1} & 0 & 0 & 1 \end{array}} \right]$$

The third step is to find the transpose of the matrix [*Q*] that satisfies [Inc]^T^[*Q*] = [0]. For the example case study, [*Q*]^T^ = [−1 −1 1 1]. The fourth step is to create the system’s freedom-topology matrix, [FT], by populating [*Q*]^T^ with the appropriately ordered twist vectors, **T**_*j*_, that mathematically model the rotational DOFs at their corresponding joints^36^. For the example case study, $$\left[ {\mathrm{FT}} \right] = \left[ {\begin{array}{*{20}{c}} { - {\mathbf{T}}_{\mathrm{1}}} & { - {\mathbf{T}}_{\mathrm{2}}} & {{\mathbf{T}}_{\mathrm{3}}} & {{\mathbf{T}}_{\mathrm{4}}} \end{array}} \right]$$. Each twist vector within this and other 2D CRAM freedom-topology matrices can be constructed using $${\mathbf{T}}_j = \left[ {\begin{array}{*{20}{c}} {{\mathbf{n}}_j} & {{\mathbf{l}}_j \times {\mathbf{n}}_j} \end{array}} \right]^T$$ where $${\mathbf{n}}_j = \left[ {\begin{array}{*{20}{c}} 0 & 0 & 1 \end{array}} \right]$$ and **l**_*j*_ is a vector that points from the global coordinate system to the corresponding cam pair’s pitch point through which its twist’s rotational axis passes. The example vector, **l**_3_, in Supplementary Fig. 4a is $${\mathbf{l}}_{\mathrm{3}} = \left[ {\begin{array}{*{20}{c}} {(R_{\mathrm{p}}/\sqrt 2 )} & {(3R_{\mathrm{p}}/\sqrt 2 )} & 0 \end{array}} \right]$$. The fifth and final step is to identify how many independent vectors, **X**, result from the null space of the system’s freedom-topology matrix (i.e., [FT]**X** = 0). This number of independent vectors is the number of CRAM DOFs. For the example case study, the number of CRAM DOFs is 1. The approach introduced here was used to generate the plots shown in Supplementary Fig. 4b-e for various lattice tessellations as they grow in size according to the patterns and boundary configurations shown. Figure 2f was generated by combining these four plots. The approach of this section is also used by the MATLAB design tool introduced in the main body of the paper to count the number of DOFs achieved by each CRAM designed.

### Prototype fabrication details

The macroscale prototypes of this paper were cut from Teflon sheets using a Trotec Speedy 100 laser cutter. The sheets were bolted to thicker sheets of acrylic at various locations to ensure that the Teflon sheets remained as flat as possible while they were cut. The cut-out layers were then assembled and joined together by hand using nuts and bolts. The connector pieces, shown in Fig. 3e, were 3D printed as hollow parts using a Stratasys uPrint SE Plus. They were also joined to their CRJs using nuts and bolts.

### Strap configuration analysis

The plots of Fig. 4d, e were generated using analytical expressions that model how a single layer of neighboring cams wrapped using the second configuration of Fig. 4b, c behave as they are rotated. As both cams rotate, the radii of their pitch circles (i.e., *R*_p1_ and *R*_p2_) would be compelled to change different amounts due to the cam wedges. These radii can be determined using the labeled parameters in Fig. 4b, c. The radius of Cam 1’s pitch circle, *R*_p1_(Φ_1_), as a function of how much Cam 1 rotates, Φ_1_, from its starting position is $R_{{\mathrm{p}}1}\left( {\Phi _1} \right) = Z_1 + \left( {t/(2\cos (\Theta _1))} \right)$, where $Z_1 = a_1\sin (\Phi _1 - \Theta _1)/\sin (\Theta _1)$, $\Theta _1 = \sin ^{ - 1}\left( {(a_1\sin ({\mathrm{\pi }} - \Phi _1))/(a_1 + R_{{\mathrm{b1}}} + t)} \right)$, and $a_i = t\left( {1 + (t/(2R_{{\mathrm{b}}i}))} \right)$ for *i* = 1 and 2. The radius of Cam 2’s pitch circle, *R*_p2_(Φ_2_), as a function of how much Cam 2 rotates, Φ_2_, from the same starting position is $R_{{\mathrm{p2}}}\left( {\Phi _2} \right) = Z_2 + \left( {t/(2\cos (\Theta _2))} \right)$, where $Z_2 = \left( {(R_{{\mathrm{b}}2} + a_2)/\sin (({\mathrm{\pi }}/2) + \Phi _2)} \right)\sin \left( {({\mathrm{\pi }}/2) - \Phi _2 - \Theta _2} \right)$ and $\Theta _2 = \sin ^{ - 1}\left( {(a_2\sin (({\mathrm{\pi }}/2) + \Phi _2))/(a_2 + R_{{\mathrm{b}}2})} \right)$. Thus, to determine how much Cam 1 has attempted to rotate, Φ_1o_, for a given rotation of Cam 2, Φ_2o_, from their starting position shown in Fig. 4c, the following equation must be solved

2 $${\int}_0^{\Phi _{1{\mathrm{o}}}} {R_{{\mathrm{p}}1}\left( {\Phi _1} \right){\mathrm{d}}\Phi _1 = } {\int}_0^{\Phi _{2{\mathrm{o}}}} {R_{{\mathrm{p}}2}\left( {\Phi _2} \right){\mathrm{d}}\Phi _2}$$

The plot shown in Fig. 4d was generated by solving Eq. (2) for cams within a single square-tessellated CRAM layer that possess the same base-circle radius (i.e., *R*_b1_ = *R*_b2_ = *R*_b_) and are wrapped using the second configuration of Fig. 4b, c for different base-circle-radius-to-strap-thickness ratios (i.e., *R*_b_/*t*). The same conditions were applied to generate the plot in Fig. 4e, which shows how the normalized distance between the centers of each cam’s base circle will be compelled to change (i.e., the distance between these centers divided by the distance between them at their starting position) as a function Cam 2’s rotation, Φ_2_, for different base-circle-radius-to-strap-thickness ratios. This normalized distance is $\left( {R_{{\mathrm{p}}1}\left( {\Phi _{1{\mathrm{o}}}} \right) + R_{{\mathrm{p}}2}\left( {\Phi _{2{\mathrm{o}}}} \right)} \right)/\left( {R_{{\mathrm{p1}}}\left( 0 \right) + R_{{\mathrm{p}}2}\left( 0 \right)} \right)$.

### Experimental validation and finite element verification of CRJ theory

Details pertaining to the fabricated CRJ of Fig. 6a, how data was collected from the CRJ, and how the FEA of the CRJ was performed are all provided in this section. The CRJ of Fig. 6a was made of Teflon with a compressive Young’s modulus of *E*_c_ = 0.27 GPa, a tensile Young’s modulus of *E*_t_ = 0.55 GPa, a compressive yield strength of *σ*_y,c_ = 24 MPa, a tensile yield strength of *σ*_y,t_ = 27 MPa, a Poisson’s ratio of *ν* = 0.46, a static coefficient of friction of *μ* = 0.07, and a density of *ρ* = 2159 kg m^−3^. All four layers were fabricated with the geometric parameters, *W* = 3.175 mm, *R*_b1_ = *R*_b2_ = 50 mm, *β*_1_ = *β*_2_ = 0.2 rad, *α*_1_ = *α*_2_ = 0.25 rad, and *t* = 0.6 mm, labeled in Fig. 5e. Each layer’s strap was fabricated slightly too short (i.e., by a length of Δ) to fit around the circular cams. The amount that these straps were stretched to fit around the cams once the joint was assembled was Δ = 0.5 mm. Multiple sets of data were immediately collected after the CRJ was assembled using the test set-up of Fig. 6d for both tension and then compression loading scenarios. The results of these tests are shown in Fig. 6f, g. The measurements were averaged for each scenario and shaded error regions were calculated to define upper and lower bounds that represent two standard deviations above and below the average measurements observed. The corresponding analytical predictions and FEA results, also shown in Fig. 6f, g, were generated using the CRJ properties and parameters specified previously. We observed that, after the tension and compression tests had been conducted, the CRJ’s straps had all stress relaxed to the extent that their relaxed length was its original length plus Δ. Thus, when we then performed the shear test shown in Fig. 6e, we compared its results with the analytical predictions and FEA results generated using the same joint properties and parameters but with Δ = 0 mm. The angular stiffness test of Fig. 6b was the last to be conducted, so it’s predicted angular stiffness of zero was also calculated using Δ = 0 mm. All FEA results were generated using the auxiliary sweep feature in COMSOL using nonlinear large-deformation settings.

### Microfabrication machine details

The subcomponents that constitute our microfabrication system are labeled with abbreviations in the schematic of Fig. 8a. The system uses a ×100 oil immersion microscope objective (MO, Olympus Plan Apo Lambda, NA = 1.45) and a three-axis micro-positioning stage (Thorlabs MAX341 and BSC203). The SHOT portion of the system consists of a 532-nm continuous wave laser (Laser Quantum Opus 3W), a 256-by-256 pixel phase-only spatial light modulator (Boulder Nonlinear Systems HSP0532–256), a two-axis scanning mirror galvanometer (Galvo, Thorlabs GVS012 and National Instruments USB-6001), a 3× beam expander (Thorlabs BE02-05-A), a half-wave plate (HWP), a beam block (BB), a polarizing beamsplitter (PBS), a mirror (M), two 4-F telescopes with doublet lenses (L1 and L2, *f* = 250–mm; L3, *f* = 100 mm; L4, *f* = 125 mm), and a shortpass dichroic mirror (DM1, λc = 567 nm). The system’s imaging column consists of a collimated 617-nm LED illumination source, two cameras (Thorlabs DCC1545M) at ×20 and ×100 magnification, beamsplitters (BS1, 50:50 R:T; BS2, 90:10 R:T), dichroic filters (ND 6.0 at 532 nm and 690–1040 nm), tube lens (Thorlabs ITL200), and doublet lenses (L5, *f* = 100 mm; L6, *f* = 60 mm). The 2PS portion of the system consists of a femtosecond laser (Spectra-Physics MaiTai eHP DS), an acousto-optic modulator (IntraAction ATM-802DA2 and ME-820-6), a 2D scanning mirror galvanometer (Galvo, Thorlabs GVS012 and National Instruments NI-9263), a HWP, two BBs, a PBS, two Ms, a quarter-wave plate (QWP), a D-shaped half mirror, doublet lenses (L7, *f* = 100 mm; L8, *f* = 50 mm; L9, *f* = 200 mm; L10 and L11, *f* = 60 mm), and a longpass dichroic mirror (DM2, λc = 650 nm).

### Code availability

The Supplementary Software 1 code is available using a GitHub repository link provided below. Additional code used to generate the plots in the paper beyond that found in Supplementary Software 1 are available from the corresponding author upon request.

(https://github.com/jonathanbhopkins/Compliant-Rolling-contact-Architected-Materials-for-Shape-Reconfigurability.git)

## Electronic supplementary material

Supplementary Information

Description of Additional Supplementary Files

Supplementary Movie 1

Supplementary Movie 2

Supplementary Movie 3

Supplementary Software 1

## Data Availability

The authors declare that all data supporting the findings of this study are included in the main manuscript file or Supplementary Information or are available from the corresponding author upon request.

## References

[1] Bertoldi K, Vitelli V, Christensen J, van Hecke M (2017). Flexible mechanical metamaterials. Nat. Rev. Mater..

[2] Grima JN, Evans KE (2000). Auxetic behavior from rotating squares. J. Mater. Sci. Lett..

[3] Lakes RS (2017). Negative-Poisson's-ratio materials: auxetic solids. Annu. Rev. Mater. Res..

[4] Kane CL, Lubensky TC (2013). Topological boundary modes in isostatic lattices. Nat. Phys..

[5] Overvelde JTB (2016). A three-dimensional actuated origami-inspired transformable metamaterial with multiple degrees of freedom. Nat. Commun..

[6] Filipov ET, Tachi T, Paulino GH (2015). Origami tubes assembled into stiff, yet reconfigurable structures and metamaterials. Proc. Natl. Acad. Sci..

[7] Kamrava S, Mousanezhad D, Ebrahimi H, Ghosh R, Vaziri A (2017). Origami-based cellular metamaterial with auxetic, bistable, and self-locking properties. Sci. Rep..

[8] Sussman DM (2015). Algorithmic lattice kirigami: a route to pluripotent materials. Proc. Natl. Acad. Sci..

[9] Neville RM, Scarpa F, Pirrera A (2016). Shape morphing Kirigami mechanical metamaterials. Sci. Rep..

[10] Rafsanjani A, Pasini D (2016). Bistable auxetic mechanical metamaterials inspired by ancient geometric motifs. Extrem. Mech. Lett..

[11] Shim J, Perdigou C, Chen ER, Bertoldi K, Reis PM (2012). Buckling-induced encapsulation of structured elastic shells under pressure. Proc. Natl. Acad. Sci..

[12] Kang SH (2014). Complex ordered patterns in mechanical instability induced geometrically frustrated triangular cellular structures. Phys. Rev. Lett..

[13] Haghpanah B, Salari-Sharif L, Pourrajab P, Hopkins J, Valdevit L (2016). Architected materials: multistable shape-reconfigurable architected materials. Adv. Mater..

[14] Jeanneau, A., Herder, J., Laliberté, T. & Gosselin, C. A compliant rolling contact joint and its application in a 3-DOF planar parallel mechanism with kinematic analysis. In *ASME 28th Biennial Mechanisms and Robotic Conference* 689–698 (American Society of Mechanical Engineers (ASME), Salt Lake City, Utah, 2004).

[15] Cannon, J. R., Lusk, C. P. & Howell, L. L. Compliant rolling-contact element mechanisms. In *ASME 29th Mechanics and Robotic Conference* 3–13 (American Society of Mechanical Engineers (ASME), Long Beach, California, 2005).

[16] Jacob's ladder, *Sci. Am.* **61**, 223–238 (1889).

[17] Wilkes, D. F. Roller-band devices. US Patent US3452309A (1969).

[18] Cadman RV (1969). Rolamite-geometry and force analysis. J. Eng. Ind..

[19] Hillberry, B. M. & Hall, A. S. Jr. Rolling contact joint. US Patent 3932045A (1976).

[20] Kuntz, J. P. *Rolling Link Mechanisms.* PhD thesis, Delft Univ. of Technology (1998).

[21] Jobin, J.-P., Buddenberg, H. S. & Herder, J. L. An underactuated prosthesis finger mechanism with rolling joints. In *ASME 28th Biennial Mechanisms and Robotic Conference* 549–559 (American Society of Mechanical Engineers (ASME), Salt Lake City, Utah, 2004).

[22] Thornton AW, Predecki P (1973). Design considerations in a rolamite knee joint prosthesis. Biomed. Mater. Symp..

[23] Herder JL, Horward MJ, Sjoerdsma W (1997). A laparoscopic grasper with force perception. Minim. Invasive Ther. Allied Technol..

[24] Te Riele, F. L. & Herder, J. L. Perfect static balance with normal springs. In *Proc. ASME Design Engineering Tech. Conferences* (American Society of Mechanical Engineers (ASME), Pittsburgh, Pennsylvania, 2001).

[25] Nelson TG, Lang RJ, Magleby SP, Howell LL (2016). Curved-folding-inspired deployable compliant rolling-contact element (D-CORE). Mech. Mach. Theory.

[26] Watt, A. M. & Pellegrino, S. Tape-spring rolling hinges. In *36th Aerospace Mechanisms Symposium* 1–14 (The Aerospace Mechanisms Symposium (AMS), Cleveland, Ohio, 2002).

[27] Meeussen AS, Paulose J, Vitelli V (2016). Geared topological metamaterials with tunable mechanical stability. Phys. Rev. X.

[28] Porter MA, Kevrekidis PG, Daraio C (2015). Granular crystals: nonlinear dynamics meets materials engineering. Phys. Today.

[29] Halverson PA, Howell LL, Magleby SP (2010). Tension-based multi-stable compliant rolling-contact elements. Mech. Mach. Theory.

[30] Halverson, P. A. *Multi-Stable Compliant Rolling-Contact Elements*. PhD thesis, Brigham Young Univ. (2007).

[31] Montierth JR, Todd RH, Howell LL (2011). Analysis of elliptical rolling contact joints in compression. J. Mech. Des..

[32] Kim, S.-H., In, H., Song, J. R. & Cho, K. J. Force characteristics of rolling contact joint for compact structure. In *IEEE 6th International Conference of Biomedical Robotics and Biomechatronics*. 1207–1212 (Institute of Electrical and Electronics Engineers (IEEE), Singapore, Singapore, 2016).

[33] Park SH, Yang DY, Lee KS (2009). Two-photon stereolithography for realizing ultraprecise three-dimensional nano/microdevices. Laser Photonics Rev..

[34] Shaw LA, Spadaccini CM, Hopkins JB (2017). Scanning holographic optical tweezers. Opt. Lett..

[35] Dawood F, Qin S, Li L, Lin EY, Fourkas JT (2012). Simultaneous microscale optical manipulation, fabrication and immobilisation in aqueous media. Chem. Sci..

[36] Sun F, Hopkins JB (2017). Mobility and constraint analysis of interconnected hybrid flexure systems via screw algebra and graph theory. J. Mech. Robot..

