# Supplementary material for: Compliant rolling-contact architected materials for shape reconfigurability
Source: Nat Commun. 2018 Nov 2;9:4594. doi: 10.1038/s41467-018-07073-5 (PMC6214902; doi:10.1038/s41467-018-07073-5)
Supplement: Supplementary file 1 — Supplementary Information [file 41467_2018_7073_MOESM1_ESM.pdf]

Compliant Rolling-contact Architected Materials for Shape Reconfigurability

Shaw et al.

Supplementary Information

Compliant Rolling-contact Architected Materials for Shape Reconfigurability

Lucas A. Shaw[1], *Samira Chizari*[1], *Matthew Dotson*[1], *Yuanping Song*[1], *Jonathan B. Hopkins*[1]*

Supplementary Software 1

The design tool consists of three MATLAB files. The master file, CRAMtool.m, contains the design tool's functions. The file, CRAMtool.fig, contains the content necessary to launch the graphical user interface (GUI). The file, images.mat, contains various images required for the GUI. Please click on the following URL or paste the URL directly into a browser to download the three required files. Once they have been downloaded, put them all into the same folder on your computer and click on the CRAMtool.m file to open it with MATLAB. Once the file is open, run it to launch the tool.

(https://github.com/jonathanbhopkins/Compliant-Rolling-contact-Architected-Materials-for-Shape-Reconfigurability.git)

Supplementary Note 1: Angular stiffness and maximum stress of ideal CRJs

This section analytically proves that CRJs would achieve zero angular stiffness and would not increase in their internal stress as they are rotated over their full range if their straps (i) are fabricated to be perfectly straight with no variation in their geometry or constituent properties, (ii) are deformed over perfectly circular cylinders, (iii) are the exact length required to prevent them from becoming loose or being stretched when the joints are assembled, and (iv) successfully guide their cylinders with perfect rolling-contact motion without stretching or slipping.

[1]Mechanical and Aerospace Engineering, University of California, Los Angeles, Los Angeles, California 90095 USA
*Corresponding author, (email: hopkins@seas.ucla.edu)

Consider a CRJ that consists of alternating layers like the layer shown in Supplementary Fig. 1. Suppose that the CRJ's straps are fabricated to be the exact length, L_{exact}, required to wrap around their corresponding cams without becoming sloppy or needing to be stretched when the joint is assembled. This length is defined by

$$L_{exact} = \left(R_{b1} + \frac{t}{2}\right)\left(\frac{\pi}{2} - \beta_1\right) + \left(R_{b2} + \frac{t}{2}\right)\left(\frac{\pi}{2} - \beta_2\right), \quad (1)$$

where R_{b1} is the base-circle radius of Cam 1, t is the thickness of the strap, β_1 is the fixed angle that defines where the strap attaches to Cam 1 as labeled in Supplementary Fig. 1, R_{b2} is the base-circle radius of Cam 2, and β_2 is the fixed angle that defines where the strap attaches to Cam 2. As long as the CRJ's straps are fabricated with this length (i.e., L_{exact}), the total strain energy, $U_{joint}(\Phi_2)$, stored in the joint as a function of how much Cam 2 has rotated, Φ_2, from its originally assembled angular position relative to Cam 1 is given by

$$U_{joint}(\Phi_2) = (O/2)\left(U_{layer}(\Phi_2) + U_{layer}(-\Phi_2)\right), \quad (2)$$

where O is the number of layers that constitute the joint and alternate according to the pattern shown in Fig. 1b, and $U_{layer}(\Phi_2)$ is the total strain energy stored in each individual layer. This strain energy is defined with respect to the coordinate system shown in Supplementary Fig. 1 as

$$U_{layer}(\Phi_2) = \frac{EWt^3}{24}\left(\int_{\psi}^{\pi-\beta_1} \frac{1}{R_{p1}(\phi_1)} d\phi_1 + \int_{\psi-\pi}^{\Phi_2-\beta_2} \frac{1}{R_{p2}(\phi_2)} d\phi_2\right), \quad (3)$$

where E is the Young's modulus of the layer's constituent material, W is the layer's out-of-plane thickness, ψ is the angle between the x-axis and the line that connects the centers of both cams, $R_{pi}(\phi_i)$ is the pitch radius of Cam i defined as $R_{pi}(\phi_i)=R_{bi}(\phi_i)+(t/2)$, and $R_{bi}(\phi_i)$ is the base-circle radius of Cam i as a function of the angle ϕ_i, labeled in Supplementary Fig. 1, where i is either 1 or 2 corresponding to either Cam 1 or Cam 2. Note that supplementary equation (2) and (3) are general for CRJs with cams of any shape—not just circles. Supplementary equation (3) was derived[1] under the assumption that the layer's strap is

initially fabricated straight with no stored strain energy and possesses no variation in its thickness, width, or material properties along its length. For CRJs consisting of perfectly circular cams like the one shown in Supplementary Fig. 1, supplementary equation (3) simplifies to

$$U_{\text{layer}}(\Phi_2)=\frac{EWt^3}{24}\left(\frac{\pi-\beta_1-\psi}{R_{\text{p1}}}+\frac{\Phi_2-\beta_1-\psi+\pi}{R_{\text{p2}}}\right), \tag{4}$$

because $R_{bi}(\phi_i)$ would remain constant as ϕ_i varies. If the straps within each layer perfectly enforce rolling-contact kinematics so that the cams are not permitted to slip as they rotate according to

$$\psi=\left(\frac{R_{\text{p2}}}{R_{\text{p1}}+R_{\text{p2}}}\right)\Phi_2+\frac{\pi}{2}, \tag{5}$$

supplementary equation (4) reduces to

$$U_{\text{layer}}(\Phi_2)=\frac{EWt^3}{24}\left(\frac{(\pi/2)-\beta_1-\Phi_2}{R_{\text{p1}}}+\frac{(\pi/2)-\beta_2+\Phi_2}{R_{\text{p2}}}\right). \tag{6}$$

If supplementary equation (6) is used in conjunction with supplementary equation (2), the total strain energy, $U_{\text{joint}}(\Phi_2)$, stored in the CRJ as a function of Φ_2 is found to be

$$U_{\text{joint}}(\Phi_2)=\frac{OEWt^3}{48}\left(\frac{\pi-2\beta_1}{R_{\text{p1}}}+\frac{\pi-2\beta_2}{R_{\text{p2}}}\right). \tag{7}$$

Note that this strain energy remains constant as Cam 2 rotates to various angular positions (i.e., as Φ_2 changes) and thus no moment is required to rotate the joint. We have, therefore, proven that CRJs will achieve zero rotational stiffness if their straps satisfy the ideal conditions specified at the beginning of this section.

The maximum stress, $\sigma_{\text{max}}(\Phi_2)$, experienced by CRJs that satisfy the same ideal conditions is $\sigma_{\text{max}}(\Phi_2)=Et/(2R_{\text{ps}})$, where R_{ps} is equal to whichever cam's pitch radius, $R_{\text{p}i}$, is the smallest. Thus, unlike other traditional compliant joints (e.g., Fig. 1a) that increase in stress as they rotate, the maximum stress within ideal CRJs remains constant as Φ_2 changes

over the joint's full range of motion. Thus, the ranges of CRJs are not limited by the yield strength of their constituent materials.

Although the derivations of this section assume ideal conditions, the general theory that governs how CRJs behave without such assumptions for any loading scenario is provided in Supplementary Note 2.

Supplementary Note 2: Modeling the behavior of CRJs

This section provides the analytical theory required to model the full nonlinear behavior of general CRJs for any loading scenario. The geometric parameters used to model the behavior of CRJs are labeled in Fig. 5e. The CRJ strap thickness is t, the base-circle radii of the CRJ's cams for i=1 or 2 are R_{bi}. The angles between the lines defined by where the straps attach to their cams and the horizontal lines that pass through the centers of the cams after they are assembled are β_i. The gap distances between the straps and the parallel features cut from corresponding cams to facilitate fabrication feasibility are δ_i. The number of strap-thickness lengths, t, over which the straps attach to the perimeter of their cams' base circles is C. The angles between the pair of lines that intersect the centers of the cams at either end of the cams' parallel fabrication features are α_i. These angles are defined according to $\alpha_i = \cos^{-1}\left((R_{bi}-\delta_i)/R_{bi}\right)$. The fabricated lengths of the CRJ's straight straps are L_o=L-Δ, where L is the length of the straps once the CRJ is assembled and Δ is the amount that the straps are stretched during assembly. The assembled length, L, is defined according to

$$L = 2R_{p1}\sin\left(\alpha_1/2\right) + R_{p1}\left((\pi/2)-\alpha_1-\beta_1\right) + 2R_{p2}\sin\left(\alpha_2/2\right) + R_{p2}\left((\pi/2)-\alpha_2-\beta_2\right), \quad (8)$$

where R_{pi} are the pitch radii of the CRJ's cams defined by R_{pi}=R_{bi}+(t/2) for i=1 or 2. The width of each alternating layer within the CRJ is W.

We will now derive the equations that relate the relative locations (i.e., x_2 and y_2 labeled in Fig. 5(f, g)) and angular orientation (i.e., Φ_2) of Cam 2 with respect to Cam 1 to the loads

that would need to be imparted on Cam 2 to move it there. To this end, we will consider two scenarios—tension and compression.

The tension scenario, shown in Fig. 5f, occurs when $D \le 0$, where

$$D = \left(R_{p1} + R_{p2}\right) - \sqrt{x_2^2 + y_2^2}\,. \tag{9}$$

For this scenario, the angle over which the strap is bent around Cam 1, θ_1, is

$$\theta_1 = \pi - \psi - \lambda_1 - \alpha_1 - \beta_1\,, \tag{10}$$

where the angle ψ, labeled in Fig. 5a, is

$$\psi = \cos^{-1}\left(x_2 / \sqrt{x_2^2 + y_2^2}\right). \tag{11}$$

Note that the x_2 variable is shown as a negative value in Fig. 5a according to the coordinate system defined at the center of Cam 1. The angle λ_1 from supplementary equation (10), labeled in Fig. 5a, is

$$\lambda_1 = \lambda_2 = \cos^{-1}\left(R_{p1} / \left(R_{p1} + \left(R_{p1}\left(\sqrt{x_2^2 + y_2^2} - \left(R_{p1} + R_{p2}\right)\right)\Big/\left(R_{p1} + R_{p2}\right)\right)\right)\right). \tag{12}$$

The angle over which the strap is bent around Cam 2, θ_2, is

$$\theta_2 = \pi - \psi - \lambda_2 - \alpha_2 - \beta_2 + \Phi_2\,, \tag{13}$$

where the angle λ_2, labeled in Fig. 5a, is defined in supplementary equation (12), and Φ_2 is how much Cam 2 has rotated from its originally assembled position relative to Cam 1.

The tension, T, in the strap at any location within one of the CRJ's layers can be derived by applying equilibrium to an infinitely small portion of the strap curved around either cam as shown in Supplementary Fig. 2a[2]. In addition to experiencing a changing tensile force, dT, along its axis, this strap portion would experience a normal force increment, dN, from the cam around which it is bent, and a force increment, μdN, caused by the friction between the strap and the cam as shown. Note that μ is the static coefficient of friction of the CRJ's material. According to principles of force equilibrium

$$\left(T + dT\right)\cos\left(d\theta / 2\right) - T\cos\left(d\theta / 2\right) - \mu dN = 0 \tag{14}$$

and

$$dN - (T + dT)\sin(d\theta / 2) - T\sin(d\theta / 2) = 0. \tag{15}$$

Using small-angle approximations, supplementary equation (14) simplifies to $dN=dT/\mu$. By applying this equation with supplementary equation (15) and using more small-angle approximations, supplementary equation (15) simplifies to $(dT/d\theta)=T\mu$. By solving the resulting differential equation, it can be shown that the tension in the straight portion of the strap between the two cams, T_s, (i.e., the tension at point P labeled in Fig. 5f) is

$$T_s = T_{\alpha 1} e^{\mu\theta_1} = T_{\alpha 2} e^{\mu\theta_2}, \tag{16}$$

where $T_{\alpha i}$ are the tensions in the straight portions of the strap between the angles α_i on either cam for i=1 or 2, and θ_1 and θ_2 are defined in supplementary equation (10) and (13) respectively. Thus, according to supplementary equation (16)

$$T_{\alpha 1} = T_{\alpha 2} e^{\mu(\theta_2 - \theta_1)}. \tag{17}$$

The total amount that the strap is stretched, S, for a given position of Cam 2 (i.e., for certain values of x_2, y_2, and Φ_2) is

$$S = d_{\alpha 1} + d_{\alpha 2} + d_{\theta 1} + d_{\theta 2} + d_s, \tag{18}$$

where $d_{\alpha i}$ are the distances the strap is stretched in the regions between the angles α_i on either cam for i=1 or 2 according to

$$d_{\alpha i} = \left(T_{\alpha i} 2R_{pi} \sin(\alpha_i / 2)\right) \Big/ \left(E_t tW\right). \tag{19}$$

The E_t parameter in supplementary equation (19) is the tensile Young's modulus of the strap. The values $d_{\theta i}$ in supplementary equation (18) are the distances the strap is stretched in the regions bent over the cams between the angles θ_i for i=1 or 2 according to

$$d_{\theta i} = \int_0^{\theta_i} \frac{R_{pi} T_{\alpha i} e^{\mu\theta}}{E_t tW} d\theta = \frac{R_{pi} T_{\alpha i}}{E_t tW}\left(\frac{e^{\mu\theta_i} - 1}{\mu}\right). \tag{20}$$

The distance that the straight portion of the strap is stretched between the two cams, d_s, in supplementary equation (18) is

$$d_{\mathrm{s}} = \left(L_{\mathrm{in}} T_{\alpha 2} e^{\mu\theta_2}\right) \Big/ \left(E_{\mathrm{t}} t W\right), \tag{21}$$

where L_{in} is the initial length of that portion of the strap before the joint is assembled or loaded, and is defined according to

$$L_{\mathrm{in}} = L_{\mathrm{o}} - 2R_{\mathrm{p1}} \sin\left(\alpha_1 / 2\right) - 2R_{\mathrm{p2}} \sin\left(\alpha_2 / 2\right) - R_{\mathrm{p1}}\theta_1 - R_{\mathrm{p2}}\theta_2 . \tag{22}$$

The total amount that the strap is stretched, S, from supplementary equation (18) can also be defined as

$$S = L_{\mathrm{s}} - L_{\mathrm{in}}, \tag{23}$$

where L_{s} is defined as

$$L_{\mathrm{s}} = \left(R_{\mathrm{p1}} + R_{\mathrm{p2}}\right) \tan\left(\lambda_1\right). \tag{24}$$

Thus, as long as $S>0$, the tension in the straight portion of the strap on Cam 2, $T_{\alpha 2}$, between the angle α_2 can be derived by combining supplementary equation (17)-(21) and (23) according to

$$T_{\alpha 2} = \frac{E_{\mathrm{t}} t W \left(L_{\mathrm{s}} - L_{\mathrm{in}}\right)}{2R_{\mathrm{p2}} \sin\left(\frac{\alpha_2}{2}\right) + R_{\mathrm{p2}} \left(\frac{\left(e^{(\mu\theta_2)} - 1\right)}{\mu}\right) + \left(2R_{\mathrm{p1}} \sin\left(\frac{\alpha_1}{2}\right) + R_{\mathrm{p1}} \left(\frac{\left(e^{(\mu\theta_1)} - 1\right)}{\mu}\right)\right) e^{\mu(\theta_2 - \theta_1)} + L_{\mathrm{in}} e^{(\mu\theta_2)}}, \tag{25}$$

where L_{s} is given in supplementary equation (24) and L_{in} is given in supplementary equation (22). Note that $T_{\alpha 2}=0$ if $S \le 0$.

The tension, $T_{\alpha 2}$, is the magnitude of three forces, shown blue in Fig. 5f, that the strap imparts on Cam 2 at its position defined by x_2, y_2, and Φ_2. One of these forces acts at point O_{A} and the other two forces act at point O_{B}. The effects of two of these forces cancel because they are equal in magnitude and point in opposite directions along the same line of action. The remaining force acts on Cam 2 at point O_{B}, and its component along the x-axis, F_{OBx}, is given by

$$F_{\mathrm{OBx}} = T_{\alpha 2} \sin\left(\Upsilon\right), \tag{26}$$

where

$$\Upsilon = \Phi_2 - \beta_2 - \alpha_2 . \tag{27}$$

The component of the same force along the y-axis, F_{OBy}, is given by

$$F_{\mathrm{OBy}} = -T_{\alpha 2} \cos\left(\Upsilon\right) . \tag{28}$$

The moment, M_{OBz}, resulting from this force acting at point O_{B} on Cam 2 about the coordinate system shown in Fig. 5f is

$$M_{\mathrm{OBz}} = F_{\mathrm{OBy}} \left(x_2 + R_{\mathrm{p2}} \cos\left(\Upsilon\right)\right) - F_{\mathrm{OBx}} \left(y_2 + R_{\mathrm{p2}} \sin\left(\Upsilon\right)\right) . \tag{29}$$

The sum of the normal force increments, dN, shown green in Fig. 5f, from the strap on Cam 2 can be determined by recognizing that supplementary equation (15) simplifies to $dN=Td\theta$ using small-angle approximations. Thus, the component of force along the x-axis, F_{Nx}, that results from summing these increments together can be derived using this equation and supplementary equation (16) according to

$$F_{\mathrm{Nx}} = -\int_{\Upsilon-\theta_2}^{\Upsilon} T_{\alpha 2} e^{\mu(\Upsilon-\zeta)} \cos\left(\zeta\right) d\zeta = -\left(\frac{2T_{\alpha 2} e^{\mu\Upsilon}}{1+\mu^2}\right)\left(\left(\frac{\sin\left(\Upsilon\right) - \mu \cos\left(\Upsilon\right)}{2e^{\mu\Upsilon}}\right) - \left(\frac{\sin\left(\Upsilon-\theta_2\right) - \mu \cos\left(\Upsilon-\theta_2\right)}{2e^{\mu\left(\Upsilon-\theta_2\right)}}\right)\right), \tag{30}$$

where ζ is defined in Fig. 5f. The component of the force along the y-axis, F_{Ny}, that results from summing the same increments together can be derived similarly according to

$$F_{\mathrm{Ny}} = -\int_{\Upsilon-\theta_2}^{\Upsilon} T_{\alpha 2} e^{\mu(\Upsilon-\zeta)} \sin\left(\zeta\right) d\zeta = -\left(\frac{2T_{\alpha 2} e^{\mu\Upsilon}}{1+\mu^2}\right)\left(\left(\frac{-\mu \sin\left(\Upsilon\right) - \cos\left(\Upsilon\right)}{2e^{\mu\Upsilon}}\right) - \left(\frac{-\mu \sin\left(\Upsilon-\theta_2\right) - \cos\left(\Upsilon-\theta_2\right)}{2e^{\mu\left(\Upsilon-\theta_2\right)}}\right)\right). \tag{31}$$

The moment, M_{Nz}, resulting from this force on Cam 2 about the coordinate system shown in Fig. 5f is

$$M_{\mathrm{Nz}} = x_2 F_{\mathrm{Ny}} - y_2 F_{\mathrm{Nx}} . \tag{32}$$

The sum of the force increments, μdN, shown purple in Fig. 5a, from the friction between the strap and Cam 2 on Cam 2 can be similarly calculated. The component of force along the x-axis, F_{fx}, that results from summing these friction increments together is

$$F_{\mathrm{fx}} = \int_{\Upsilon-\theta_2}^{\Upsilon} \mu T_{\alpha 2} e^{\mu(\Upsilon-\zeta)} \sin\left(\zeta\right) d\zeta = -\mu F_{\mathrm{Ny}} . \tag{33}$$

The component of the force along the y-axis, F_{fy}, that results from summing the same friction increments together can be derived similarly according to

$$F_{\mathrm{fy}} = -\int_{\Upsilon-\theta_2}^{\Upsilon} \mu T_{\alpha 2} e^{\mu(\Upsilon-\zeta)} \cos(\zeta)\, d\zeta = \mu F_{\mathrm{Nx}} \,. \tag{34}$$

The moment, M_{fz}, resulting from this force on Cam 2 about the coordinate system shown in Fig. 5f is

$$M_{\mathrm{fz}} = x_2 F_{\mathrm{fy}} - y_2 F_{\mathrm{fx}} - R_{\mathrm{b2}} T_{\alpha 2} \left(e^{\mu\theta_2} - 1 \right). \tag{35}$$

There is also a pure moment, which acts on Cam 2, that is produced by the strap because it is bent. This moment can be determined by calculating the strain energy in the strap due to bending. This energy, U_{bend}, is

$$U_{\mathrm{bend}} = U_{\theta} + U_{\mathrm{OA}} + U_{\mathrm{OB}} + U_{\mathrm{OE}} + U_{\mathrm{OF}} \,, \tag{36}$$

where U_{θ} is the bending energy stored in the strap as a result of it being bent around both cams over the angles θ_i. By using a similar approach to that introduced in Supplementary Note 1, this energy is determined to be

$$U_{\theta} = \frac{EWt^3}{24} \left(\frac{\theta_1}{R_{\mathrm{p1}}} + \frac{\theta_2}{R_{\mathrm{p2}}} \right). \tag{37}$$

Since the straps within each layer will enforce rolling-contact kinematics about the point where the alternating straps crisscross (i.e., the point P labeled in Fig. 5a), we can apply

$$\psi = \left(\frac{R_{\mathrm{pp2}}}{R_{\mathrm{pp1}} + R_{\mathrm{pp2}}} \right) \Phi_2 + \frac{\pi}{2} \,, \tag{38}$$

to supplementary equation (10) and (13) to change supplementary equation (37) to

$$U_{\theta} = \frac{EWt^3}{24} \left(\frac{(\pi/2) - \lambda_1 - \alpha_1 - \beta_1 - \Phi_2}{R_{\mathrm{p1}}} + \frac{(\pi/2) - \lambda_2 - \alpha_2 - \beta_2 + \Phi_2}{R_{\mathrm{p2}}} \right), \tag{39}$$

where $R_{\mathrm{pp}i}$ from supplementary equation (38) are the distances from the centers of each Cam i to the point P, labeled in Fig. 5f. These distances are

$$R_{\mathrm{pp}i} = \left(R_{\mathrm{p}i} \sqrt{x_2^2 + y_2^2} \right) \Big/ \left(R_{\mathrm{p1}} + R_{\mathrm{p2}} \right). \tag{40}$$

The energies U_{OA} and U_{OB} from supplementary equation (36) are the bending energies that result from the tensioned strap being sharply bent at the points O_A and O_B, labeled in Fig. 5f. These energies can be calculated according to

$$U_{OA} = U_{OB} = \left(K_{hinge}/2\right)\left(\alpha_2/2\right)^2, \tag{41}$$

where K_{hinge} can be approximated as the stiffness of a compliant living hinge according to[3]

$$K_{hinge} = \left(\frac{EWt^2}{2(1+v)}\right)\left(\frac{1}{3} - 0.21\frac{t}{W}\left(1 - \frac{t^4}{12W^4}\right)\right), \tag{42}$$

where v is the Poisson's ratio of the CRJ's material. The bending energies U_{OE} and U_{OF} from supplementary equation (36) result from the tensioned strap being sharply bent at the points O_E and O_F, labeled in Fig. 5f. These energies can be calculated according to

$$U_{OE} = U_{OF} = \left(K_{hinge}/2\right)\left(\alpha_1/2\right)^2. \tag{43}$$

The pure moment, M_{m2}, that would need to be imparted on Cam 2 to counteract the moment imposed by the strap on Cam 2 due to the total bending energy in the strap, U_{bend} from supplementary equation (36) can be determined by taking the derivative of this energy with respect to the rotation of Cam 2, Φ_2, relative to Cam 1 according to

$$M_{m2} = \frac{dU_{bend}}{d\Phi_2} = \frac{EWt^3}{24}\left(\frac{1}{R_{p2}} - \frac{1}{R_{p1}}\right). \tag{44}$$

Note that although the derivatives of all of the energies in supplementary equation (36) become zero except for $dU_\theta/d\Phi_2$, we will later show that other regimes within the tension scenario exist where the bending energies at the various points O_A, O_B, O_E, and O_F would have an effect.

Thus, the total force component along the x-axis, F_{2x}, the total force component along the y-axis, F_{2y}, and the total moment, M_{2z}, that would need to be collectively imparted on Cam 2 to hold the single CRJ layer shown in Fig. 5f in static equilibrium at the location, x_2 and y_2, and the orientation Φ_2, would be

$$F_{2x} = -\left(F_{OBx} + F_{Nx} + F_{fx}\right), \tag{45}$$

$$F_{2\text{y}} = -\left(F_{\text{OBy}} + F_{\text{Ny}} + F_{\text{fy}}\right), \tag{46}$$

and

$$M_{2\text{z}} = -\left(M_{\text{OBz}} + M_{\text{Nz}} + M_{\text{fz}}\right) + M_{\text{m2}}. \tag{47}$$

To determine the total load that must be imparted on a full CRJ consisting of O alternating layers in the tension scenario depicted in Fig. 5f such that the entire joint will be in static equilibrium at the location, x_2 and y_2, and the orientation Φ_2, the loads determined using supplementary equation (45)-(47) must be calculated for each layer and summed together. To determine the loads on the layers with straps that crisscross with the straps from the layers shown in Fig. 5f, the same equations provided above can be used but where x_2=-x_2, y_2=y_2, and Φ_2=-Φ_2. The resulting loads calculated must also be changed before they are summed with the other loads such that $F_{2\text{x}}$=-$F_{2\text{x}}$, $F_{2\text{y}}$=$F_{2\text{y}}$, and $M_{2\text{z}}$=-$M_{2\text{z}}$. Note that this analysis only works if all out-of-plane loads cancel because the layers alternate according to the pattern shown in Fig. 1b and the number of layers, O, is a multiple of four. Finally, note from supplementary equation (39) and (44) that as long as the straps are bent around both cams such that θ_1 and θ_2 are greater than zero for all the layers within the CRJ, the moments from each alternating layer due to strap bending, M_{m2}, will always cancel so that the only loads imparted on Cam 2 that have any effect on the joint's stiffness are those caused by the strap being stretched.

We will now consider the compression scenario shown in Fig. 5g that occurs when D>0 (see supplementary equation (9)). For a given x_2 and y_2 that satisfy D>0, the magnitude of the compression force, F_c, imparted on Cam 2 from Cam 1 shown in Fig. 5g as well as the distance over which the cams are compressed flat, b, can both be calculated by simultaneously solving the following two equations, which are adaptations from Hertzian-contact theory[4]. These equations are

$$D = \frac{2F_\text{c}}{\pi W}\left(\frac{1-\nu^2}{E_\text{c}}\right)\left(\frac{2}{3} + \ln\left(\frac{8R_{\text{b1}}}{b}\right) + \ln\left(\frac{8R_{\text{b2}}}{b}\right)\right) + \frac{F_\text{c} t}{E_\text{c} W b} \tag{48}$$

and

$$b = 4\sqrt{\left(2F_{\mathrm{c}}\left((1-\nu^2)/E_{\mathrm{c}}\right)\right)/\left(\pi W\left((1/R_{\mathrm{b1}})+(1/R_{\mathrm{b2}})\right)\right)}\,, \tag{49}$$

where E_{c} is the compressive Young's modulus of the CRJ material. Once these values are known, the same geometric-compatibility equations used for the tension scenario (i.e., supplementary equation (10)-(13)) apply to the compression scenario except that supplementary equation (12) is replaced by

$$\lambda_i = \sin^{-1}\left(b/\left(2R_{\mathrm{p}i}\right)\right) \tag{50}$$

such that λ_1 and λ_2 are no longer equal for different-sized cams. A free body diagram of the loads acting on the straight portion of the strap that is sandwiched between the two cams of Fig. 5g is shown in static equilibrium in Supplementary Fig. 2b. Note that μF_{c} are the friction forces generated between the strap and the cams, and $M_{\mathrm{OC,bend}}$ and $M_{\mathrm{OD,bend}}$ are the bending moments imposed on the strap due to the fact that the tensioned strap is bent sharply at points O_{C} and O_{D}. The free body diagram of Supplementary Fig. 2b proves that the tension in this portion of the strap, T_{s}, remains constant over its length. Thus, the same equations used to calculate the tension in the strap at any location for the tension scenario (i.e., supplementary equation (14)-(25)) apply to the compression scenario except that supplementary equation (24) is replaced by $L_{\mathrm{s}}=b$. Furthermore, the loads imparted by the tensioned strap on Cam 2 that were derived for the tension scenario (i.e., supplementary equation (26)-(35)) apply to the compression scenario except that additional loads exist for the compression scenario.

The tension, T_{s}, is the magnitude of two forces, shown red in Fig. 5g, that the tensioned strap imparts on Cam 2 at point O_{C}. The component along the x-axis, F_{OCx}, of both of these forces summed together is given by

$$F_{\mathrm{OCx}} = -T_{\mathrm{s}}\left(\sin\left(\Upsilon-\theta_2\right)+\sin\left(\psi\right)\right). \tag{51}$$

The component of the same force along the y-axis, F_{OCy}, is given by

$$F_{\mathrm{OCy}} = T_{\mathrm{s}}\left(\cos\left(\Upsilon-\theta_2\right)+\cos\left(\psi\right)\right). \tag{52}$$

The moment, M_{OCz}, resulting from the same force acting at point O_C on Cam 2 about the coordinate system shown in Fig. 5g is

$$M_{OCz} = F_{OCy} R_{p1} \cos\left(\psi - \lambda_1\right) - F_{OCx} R_{p1} \sin\left(\psi - \lambda_1\right). \tag{53}$$

Additionally, the component of the compressive force along the *x*-axis, F_{compx}, shown black and labeled with its magnitude, F_c, in Fig. 5g is given by

$$F_{compx} = F_c \cos\left(\psi\right). \tag{54}$$

The component of the same force along the *y*-axis, F_{compy}, is given by

$$F_{compy} = F_c \sin\left(\psi\right). \tag{55}$$

Note that no moment is applied to Cam 2 by this compressive force about the coordinate system shown in Fig. 5g.

There is also a pure moment, which acts on Cam 2, that is produced by the strap because it is bent. This moment can be determined by calculating the strain energy in the strap due to bending. This energy, U_{bend}, is similar to that given in supplementary equation (36) but with two additional terms according to

$$U_{bend} = U_\theta + U_{OA} + U_{OB} + U_{OC} + U_{OD} + U_{OE} + U_{OF}. \tag{56}$$

The energy, U_θ, in supplementary equation (56) can be found by applying supplementary equation (38) with supplementary equation (10), (13), and (37) using

$$R_{ppi} = \sqrt{R_{pi}^2 - \left(b/2\right)^2} \tag{57}$$

instead of supplementary equation (40). The resulting energy is

$$U_\theta = \frac{EWt^3}{24}\left(\frac{\left(\pi/2\right) - \lambda_1 - \alpha_1 - \beta_1}{R_{p1}} + \frac{\left(\pi/2\right) - \lambda_2 - \alpha_2 - \beta_2}{R_{p2}} + \left(\frac{R_{pp1}}{R_{p2}} - \frac{R_{pp2}}{R_{p1}}\right)\left(\frac{\Phi_2}{R_{pp1} + R_{pp2}}\right)\right). \tag{58}$$

The energies U_{OA}, U_{OB}, U_{OE}, and U_{OF} in supplementary equation (56) are the same as those given in supplementary equation (41) and (43). The two new terms in supplementary equation (56), U_{OC} and U_{OD}, are the bending energies that result from the tensioned strap being sharply bent at the points O_C and O_D, labeled in Fig. 5g. These energies can be calculated according to

$$U_{\mathrm{OC}}=\left(K_{\mathrm{hinge}}/2\right)\left(\cos^{-1}\left(-\sin\left(\psi\right)\sin\left(\Upsilon-\theta_2\right)-\cos\left(\psi\right)\cos\left(\Upsilon-\theta_2\right)\right)\right)^2 \tag{59}$$

and

$$U_{\mathrm{OD}}=\left(K_{\mathrm{hinge}}/2\right)\left(\cos^{-1}\left(\sin\left(\psi\right)\sin\left(\psi+\lambda_1\right)+\cos\left(\psi\right)\cos\left(\psi+\lambda_1\right)\right)\right)^2. \tag{60}$$

Note, also that the bending moments, $M_{\mathrm{OC,bend}}$ and $M_{\mathrm{OD,bend}}$, labeled in the free body diagram of Supplementary Fig. 2b are

$$M_{\mathrm{OC,bend}}=K_{\mathrm{hinge}}\left|\cos^{-1}\left(-\sin\left(\psi\right)\sin\left(\Upsilon-\theta_2\right)-\cos\left(\psi\right)\cos\left(\Upsilon-\theta_2\right)\right)\right| \tag{61}$$

and

$$M_{\mathrm{OD,bend}}=K_{\mathrm{hinge}}\left|\cos^{-1}\left(\sin\left(\psi\right)\sin\left(\psi+\lambda_1\right)+\cos\left(\psi\right)\cos\left(\psi+\lambda_1\right)\right)\right|. \tag{62}$$

The pure moment, M_{m2}, that would need to be imparted on Cam 2 to counteract the moment imposed by the strap on Cam 2 due to the total bending energy in the strap, U_{bend} from supplementary equation (56), can be determined by taking the derivative of the energy with respect to the rotation of Cam 2, Φ_2, relative to Cam 1 according to

$$M_{\mathrm{m2}}=\frac{dU_{\mathrm{bend}}}{d\Phi_2}=\frac{EWt^3}{24}\left(\frac{1}{R_{\mathrm{pp1}}+R_{\mathrm{pp2}}}\right)\left(\frac{R_{\mathrm{pp1}}}{R_{\mathrm{p2}}}-\frac{R_{\mathrm{pp2}}}{R_{\mathrm{p1}}}\right). \tag{63}$$

Note that the derivatives of all of the energies in supplementary equation (56) become zero (even the two new terms) except for $dU_\theta/d\Phi_2$. Additionally, note from supplementary equation (58) that as long as the straps are bent around both cams such that θ_1 and θ_2 are greater than zero for all the layers within the CRJ, the moments from each alternating layer due to strap bending, M_{m2}, will always cancel for the compression scenario as well as for the tension scenario.

If the friction force, shown orange and labeled by its magnitude, μF_{c}, in Fig. 5g, is temporarily neglected, the total force component along the x-axis, $F_{2\mathrm{x}}$, the total force component along the y-axis, $F_{2\mathrm{y}}$, and the total moment, $M_{2\mathrm{z}}$, that would need to be collectively

imparted on Cam 2 to hold the single CRJ layer shown in Fig. 5g in static equilibrium at the location, x_2 and y_2, and the orientation Φ_2, would be

$$F_{2x} = -\left(F_{OBx} + F_{Nx} + F_{fx} + F_{OCx} + F_{compx}\right), \tag{64}$$

$$F_{2y} = -\left(F_{OBy} + F_{Ny} + F_{fy} + F_{OCy} + F_{compy}\right), \tag{65}$$

and

$$M_{2z} = -\left(M_{OBz} + M_{Nz} + M_{fz} + M_{OCz}\right) + M_{m2}. \tag{66}$$

To include the friction force, shown orange and labeled by its magnitude, μF_c, in Fig. 5g, within our analysis, the loads required to keep Cam 2 in static equilibrium for the case of two alternating CRJ layers need to be considered simultaneously. After summing the loads in supplementary equation (64)-(66) for two such alternating layers using the approach described at the end of the tension-scenario discussion, the direction of the resulting force along the length of the portion of the strap that is sandwiched between the two cams determines the direction of the friction force. If that component of the resulting force is positive according to the coordinate system shown in Fig. 5g, the component of the friction force acting on Cam 2 from the two layers of alternating straps along the x-axis, F_{fricx}, is

$$F_{fricx} = -2\mu F_c \sin(\psi). \tag{67}$$

The component of the same friction force along the y-axis, F_{fricy}, is

$$F_{fricy} = 2\mu F_c \cos(\psi). \tag{68}$$

The moment, M_{fricz}, resulting from the same friction force on Cam 2 about the coordinate system shown in Fig. 5g is

$$M_{fricz} = F_{fricy}\left(\sqrt{R_{p1}^2 - (b/2)^2} + (t_*/2)\right)\cos(\psi) - F_{fricx}\left(\sqrt{R_{p1}^2 - (b/2)^2} + (t_*/2)\right)\sin(\psi), \tag{69}$$

where t_* is the compressed thickness of the strap labeled in Supplementary Fig. 2b. This thickness is given by

$$t_* = t\left(1 - \left(F_c/(E_c W b)\right)\right). \tag{70}$$

If, however, the component of the resulting force discussed previously is negative according to the coordinate system shown in Fig. 5g, supplementary equation (67)-(69) should be multiplied by negative one. If the component of the force is zero, supplementary equation (67)-(69) should be multiplied by zero. Whatever the case may be, the appropriate version of supplementary equation (67)-(69) should be multiplied by negative one and summed with supplementary equation (64)-(66) applied to two alternating layers. The result should then be multiplied by $O/2$ to calculate the total load that should be imparted on the CRJ to keep it in static equilibrium at the position and orientation defined by x_2, y_2, and Φ_2.

Note that the analysis of this section applies only to the tension and compression scenarios when the straps within each CRJ layer are bent around both cams such that θ_1 and θ_2 are greater than zero. There are, however, 8 other regimes within the tension scenario, and 10 other regimes within the compression scenario where the above analysis would require minor to drastic alterations. Consider, for instance, the two alternating layers shown in Supplementary Fig. 2(c, d). The first layer shown would possess an angle θ_1=0 and the second layer shown would possess an angle θ_2=0. For a CRJ consisting of layers with cams arranged in this way, much of the conclusions deduced from the previous analysis would no longer hold true. The bending moment imposed on Cam 2 by the strap of the second layer where the strap attaches to Cam 2 would no longer be zero, for example. Moreover, the straps stop enforcing rolling-contact kinematics when their CRJ's axis of rotation, which occurs where the alternating layers of straps crisscross (i.e., point P), no longer lies on the line that connects the centers of both cams. Thus, the other regimes invoke different mechanics, which model the effects that prevent the cams from rotating with respect to each other when some of their straps have unwrapped off of their circular contours. The theory to model the mechanics of all 9 regimes in the tension scenario and all 11 regimes in the compression scenario for fully modeling a CRJ in any configuration are detailed in the MATLAB script provided.

Supplementary Note 3: CRAM software design tool

This section describes how the theory of Supplementary Note 2 was used to enable the new MATLAB design tool provided in Supplementary Software 1. Other theoretical advances that enabled the capabilities of this software are also described and cited as specific functions or lines of code within the tool. Instructions for launching the software are also provided.

The software design tool simulates the behavior of general CRAMs by applying the equations of Supplementary Note 2 to determine the local stiffness values of each constituent CRJ within the CRAM's lattice at any configuration about the CRJ's axis of rotation (i.e., point P). These stiffness values are determined by identifying how the static-equilibrium loads change for small perturbations of x_2, y_2, and Φ_2. The resulting stiffness values are organized within local stiffness matrices that are analytically stitched together to construct the CRAM's global stiffness matrix using the theory detailed in the "BuildStiffnessMatrix" function, included within the MATLAB script provided. This function also constructs local stiffness matrices where cams that are not joined together by CRJs attempt to collide so that the tool doesn't let any cams unrealistically pass through each other's geometry.

The theory necessary to calculate the pitch radius of every circular cam within a general CRAM lattice once the pitch radius of any one of those cams have been specified is provided in lines 1288-1373 of the MATLAB script. The theory necessary to calculate the strap lengths within a general CRAM design such that the straps are as long as possible to maximize the lattice's deformation range while satisfying geometric compatibility is provided in lines 1379-1506 of the MATLAB script. The theory necessary to determine if a designed CRAM will yield when it is assembled is provided in lines 1551-1575 of the MATLAB script. The theory necessary to reverse engineer a CRAM design to determine how each unassembled layer should be fabricated with straight straps is provided in the "drawlayerundeformed" function in the MATLAB script.

Supplementary References

1. Halverson, P. A. Multi-stable compliant rolling-contact elements, PhD thesis, Brigham Young University (2007).
2. Crandall, S. H., Dahl, N. C., Lardner, T. J. & Sivakumar, M. S. An Introduction to Mechanics of Solids. 2nd ed. (Tata McGraw-Hill, New Delhi, 1978).
3. Delimont, I. L., Magleby, S. P. & Howell, L. L. Evaluating Compliant Hinge Geometries for Origami-Inspired Mechanisms. J. Mech. Robot. **7**, 011009-011009-8 (2015).
4. Popov, V. L. Contact Mechanics and Friction: Physical Principles and Applications Ch. 3 (Springer, Berlin-Heidelberg, 2010).

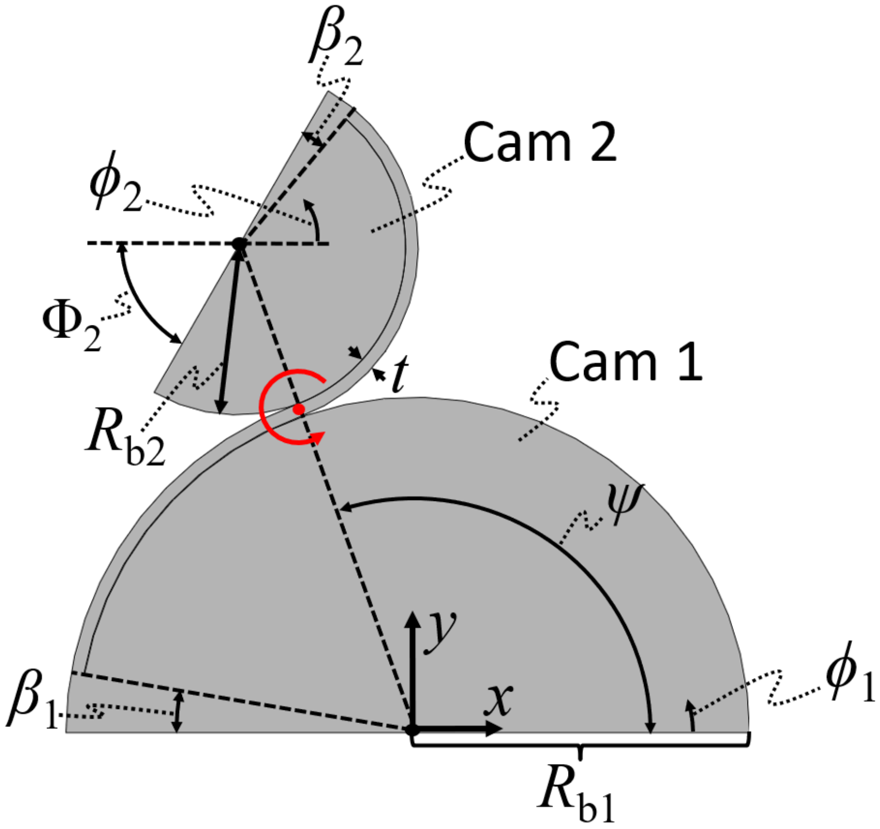

Supplementary Figure 1. Compliant rolling-contact joint (CRJ) geometric parameters. Parameters necessary to prove that the strain energy and stress of an ideal CRJ with multiple alternating layers does not change as it rotates.

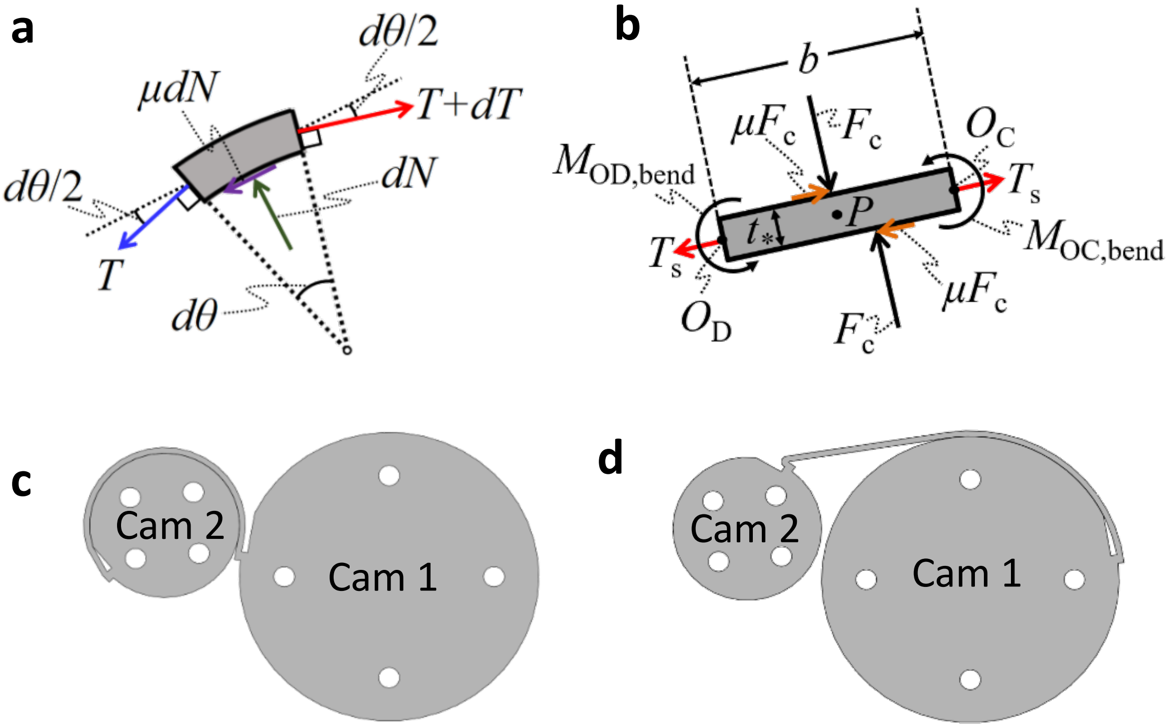

Supplementary Figure 2. Free body diagrams and compliant rolling-contact joint (CRJ) layers deformed within different regimes. a, A free body diagram of an infinitely small portion of strap wrapped around either cam. **b**, A free body diagram of the portion of the strap that is sandwiched between the flattened cams in the compression scenario shown in Fig. 5g. **c** and **d**, two alternating layer examples that constitute a single CRJ but belong within different regimes.

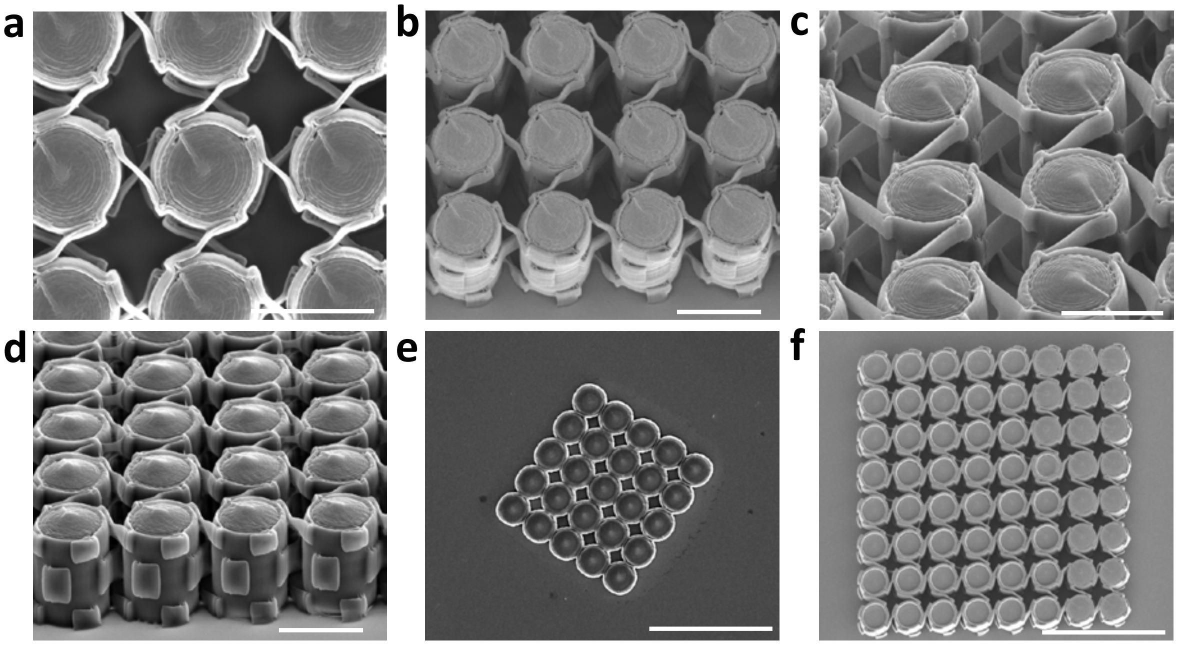

Supplementary Figure 3. Fabricated compliant rolling-contact architected material (CRAM) photos. a-**f**, Scanning-electron-microscope (SEM) images of a square-tessellated CRAM fabricated using the two-photon stereolithography (2PS) portion of our system (scale bar in **a**-**d**, 10 μm, **e**, 50 μm, **f**, 40 μm).

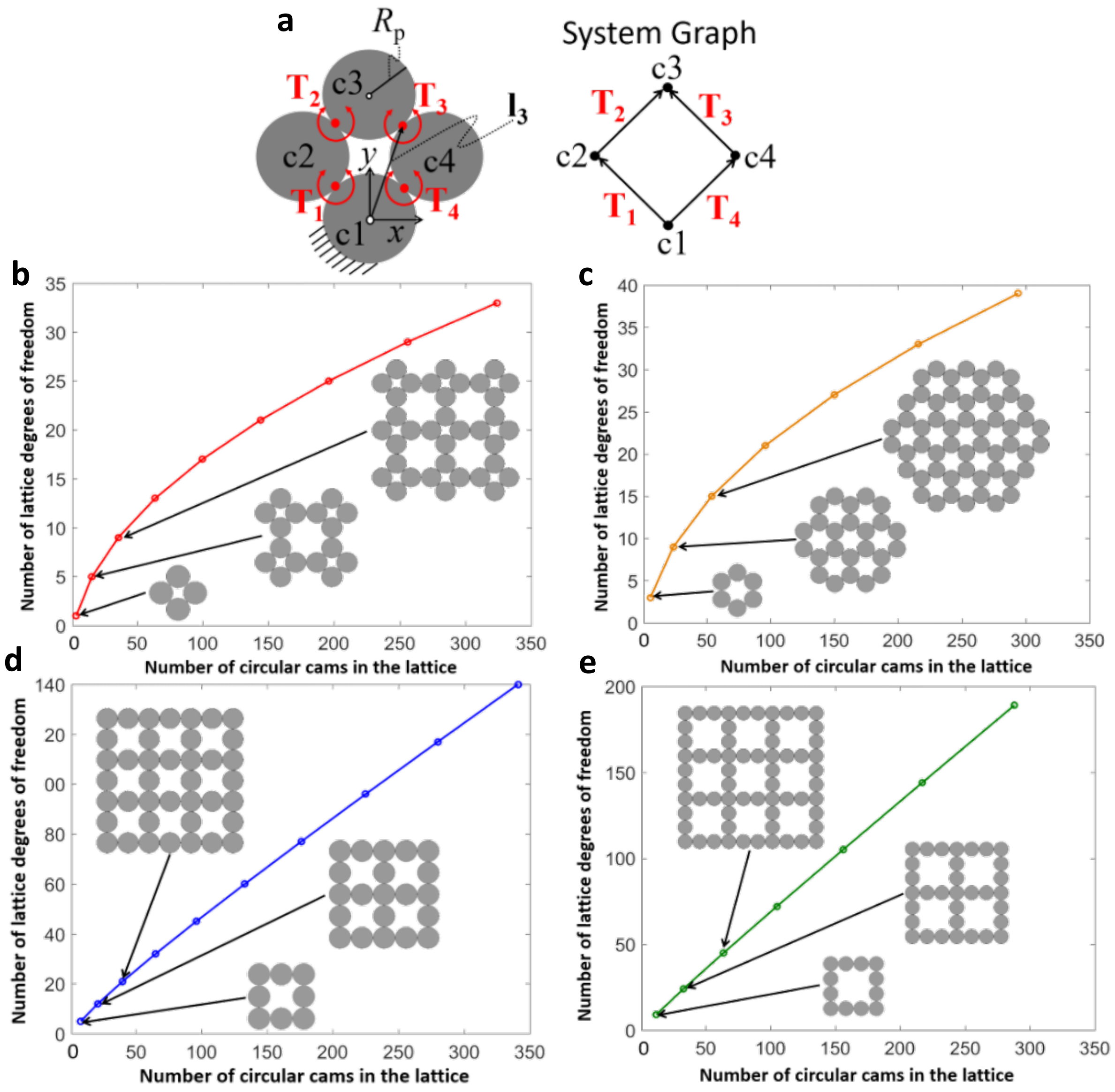

Supplementary Figure 4. Calculating the number of compliant rolling-contact architected material (CRAM) degrees of freedom (DOFs). a, Example diagram of a CRAM provided to demonstrate the approach for calculating the number of DOFs achieved by general CRAM tessellations. Plots are given to show how the number of lattice DOFs increases as the lattice size increases for **b**, a square-octagon tessellation, **c**, a hexagon tessellation, **d**, a square tessellation with one circular cam between each pair of neighboring vertices, and **e**, a square tessellation with two circular cams between each pair of neighboring vertices.
