# Supplementary material for: Compliant rolling-contact architected materials for shape reconfigurability
Source: Nat Commun. 2018 Nov 2;9:4594. doi: 10.1038/s41467-018-07073-5 (PMC6214902; doi:10.1038/s41467-018-07073-5)
Supplement: Supplementary file 2 — Description of Additional Supplementary Files [file 41467_2018_7073_MOESM2_ESM.docx]

**Title:** Supplementary Movie 1

**Description:** Video of compliant rolling-contact joint (CRJ) and compliant rolling-contact architected material (CRAM) animations and two-dimensional and three-dimensional CRAM prototype demonstrations classified into various categories.

**Title:** Supplementary Movie 2

**Description:** Video showing a real-time demo of the compliant rolling-contact architected material (CRAM) design tool as well as design case studies generated by the tool.

**Title:** Supplementary Movie 3

**Description:** Video detailing the proposed approach for fabricating compliant rolling-contact architected material (CRAM) designs at the microscale as well as live demonstrations of designs being printed using two-photon stereolithography and assembled using scanning holographic optical tweezers.

**Title:** Supplementary Software 1

**Description:** The design tool consists of three MATLAB files. The master file, CRAMtool.m, contains the design tool’s functions. The file, CRAMtool.fig, contains the content necessary to launch the graphical user interface (GUI). The file, images.mat, contains various images required for the GUI. Please click on the following URL or paste the URL directly into a browser to download the three required files. Once they have been downloaded, put them all into the same folder on your computer and click on the CRAMtool.m file to open it with MATLAB. Once the file is open, run it to launch the tool. [https://drive.google.com/open?id=1AjXTNccLgxzl7XR9Oz8ocQA4oHTNI70J]
